# Ultrafast Coupling of Optical Near Fields to Low-Energy Electrons Probed in a Point-Projection Microscope

**DOI:** 10.1021/acs.nanolett.3c00738

**Published:** 2023-06-06

**Authors:** Andreas Wöste, Germann Hergert, Thomas Quenzel, Martin Silies, Dong Wang, Petra Groß, Christoph Lienau

**Affiliations:** † Institut für Physik und Center for Nanoscale Dynamics (CeNaD), Carl von Ossietzky Universität, 26129 Oldenburg, Germany; ‡ Institut für Werkstofftechnik und Institut für Mikro- und Nanotechnologien, TU Ilmenau, 98693 Ilmenau, Germany

**Keywords:** photon-induced near-field electron microscopy, ultrafast electron microscopy, plamonic near fields, low-energy electrons, nanoantenna

## Abstract

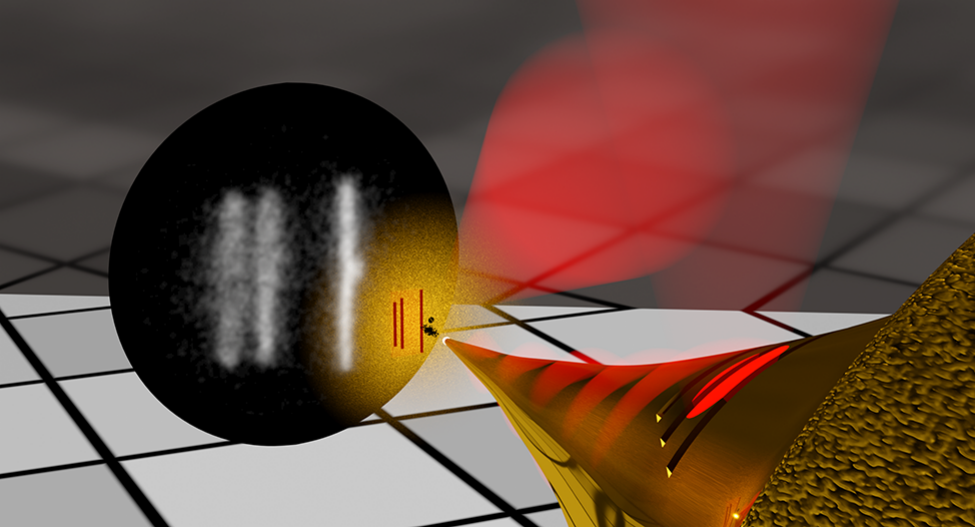

We report the first observation of the coupling of strong optical near fields to wavepackets of free, 100 eV electrons with <50 fs temporal resolution in an ultrafast point-projection microscope. Optical near fields are created by excitation of a thin, nanometer-sized Yagi-Uda antenna, with 20 fs near-infrared laser pulses. Phase matching between electrons and near fields is achieved due to strong spatial confinement of the antenna near field. Energy-resolved projection images of the antenna are recorded in an optical pump–electron probe scheme. We show that the phase modulation of the electron by transverse-field components results in a transient electron deflection while longitudinal near-field components broaden the kinetic energy distribution. This low-energy electron near-field coupling is used here to characterize the chirp of the ultrafast electron wavepackets, acquired upon propagation from the electron emitter to the sample. Our results bring direct mapping of different vectorial components of highly localized optical near fields into reach.

The coupling of free electrons to localized optical near fields enabled the development of novel types of ultrafast electron microscopes.^1,2^ Swift electrons, with kinetic energies in the 100 keV range, are readily used in photon-induced near-field electron microscopy (PINEM) to investigate localized optical near fields of nanosized systems with excellent spatial and spectral resolution.^3−10^ Full control of the quantum phase of free electron wavepackets in PINEM may be key for pushing the time resolution of ultrafast electron microscopy to the attosecond regime^11−13^ while maintaining atomic spatial resolution, enabling direct imaging of electronic processes in matter on their natural time and length scales.^4,12,14−16^

Typically employed swift electrons in transmission electron microscopes propagate at speeds between  and  of the speed of light in vacuum. When interacting with small nanostructures, with dimensions of only a few nanometers, the interaction time with the optical near field and thus the coupling efficiency to a single nanoconfined optical mode is, therefore, inherently weak.^17,18^ The use of much slower electrons, with kinetic energies of few tens or hundreds of eV, may, in principle, increase the field–electron interaction time and thus enhance the coupling.^18,19^ Compared with swift electrons, slow electrons exhibit significantly larger deflection angles^19,20^ at a given field amplitude, providing practical access to different near-field vector components. Exploring and exploiting these favorable properties of slow electrons for near-field probing, however, is experimentally challenging since efficient near-field coupling requires phase matching between the electron wave and the near field. For slow electrons, this is achieved only for nanostructures in the 10 nm range or below. So far, therefore, PINEM effects could only be resolved experimentally for electrons down to 10 keV,^21^ far above the energies typically used in ultrafast electron microscopy techniques employing low-energy electrons.^22−25^ The ability to experimentally probe the interaction of near fields with slow electrons is therefore of considerable importance for the ongoing development of ultrafast point-projection electron microscopy (UPEM).^22−28^

Here we report what we believe is the first observation of the time-resolved diffraction of slow electrons by optical near fields in a point-projection microscope. Free electron probe pulses with kinetic energies as low as 80 eV and an initial pulse duration of <20 fs, generated by plasmonic nanofocusing, interact with the near field of a nanometer-sized Yagi-Uda antenna that is strongly enhanced in comparison to that of the employed near-infrared pump pulse. We observe electron energy gain and loss due to the interaction with longitudinal near-field components and a sideways deflection by transverse components. Variations in kinetic energy distribution, arising in time-resolved optical pump–electron probe measurements, give insight into the group velocity dispersion of the electron wavepacket acquired upon propagation from the electron emitter to the sample.^29^

We probe the interaction of slow electrons with localized optical near fields in the near-infrared (NIR) range with an energy-resolved UPEM^23^ in an optical pump–electron probe experiment. The experiment is sketched in Figure 1a. NIR laser pulses with a duration of 20 fs, centered at ω_*p*_ ≈ 1 PHz (1900 nm), are delivered by a home-built NOPA system operating at 175 kHz repetition rate. Those pulses are used to generate electron probe pulses with a duration of <20 fs by plasmonic nanofocusing. The electrons are emitted in a sixth order multiphoton photoemission process from the 30 nm diameter apex of a gold nanotip (Figure 1a, Supporting Information Section 3).^30−33^ These electrons are accelerated by a bias voltage of less than 100 V to velocities of 5.3–6 nm/fs, toward a 5.8 μm distant, freestanding, 13 nm thick gold film. After ∼1 ps of propagation, they are transmitted through a Yagi-Uda type nanoresonator, written into the gold film by helium-ion-beam milling (Figure 1b). A time-delayed replica of the NIR beam is focused to a 30 μm spot size onto the film to optically excite the near fields of the antenna. At the chosen acceleration voltages, the electron transit time, *T*, through the optical near field is 3–3.4 fs, roughly one-half of an optical cycle (∼3.2 fs), such that phase-matching, *T* ≤ π/ω_*p*_, between the near field and the electron is fulfilled.^1,18,19^ This is difficult to achieve for slow electrons and requires a strong spatial localization of the optical near field.

**Figure 1:**
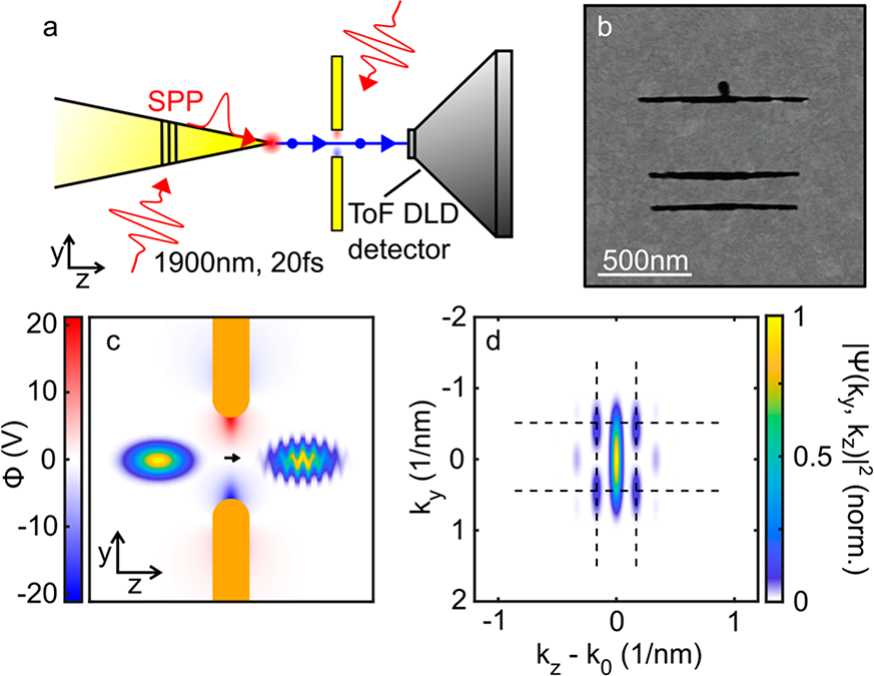
(a) Ultrashort electron pulses are photoemitted from the apex of a gold nanotip by plasmonic nanofocusing and accelerated by applying a bias voltage of 80 to 100 V between the tip and the sample. After transmission through the sample, a magnified shadow image is formed on a distant delay line detector (DLD), recording the 3D momentum distribution of the impinging electrons. (b) Scanning electron microscope image of the Yagi-Uda antenna structure, milled into a 13 nm thick, free-standing gold film. (c) Interaction of a 20 fs, 100 eV electron pulse with the near field of the Yagi-Uda antenna. The spatial profile of the electron density is shown schematically 10 fs before and 10 fs after the interaction with the optically excited sample. Electron propagation leads to an undulatory modulation of the charge density in transverse direction. (d) Momentum distribution of the electron wavepacket after the near-field interaction, showing sidebands along *k*_*z*_, the momentum component along the propagation direction, spaced by the momentum mismatch Δ*k* = ω_*p*_/*v*_0_ between the photon and the electron. These sidebands reflect the absorption and stimulated emission of multiple near-field photons by the passing electron wavepacket. The near field induced electron deflection along the transverse *y*-direction modulates the momentum distribution along *k*_*y*_.

After traversing the sample, the impact position of each electron is measured by a delay-line detector (DLD) to create a magnified point-projection image of the sample.^23^ In addition, the DLD records the electron arrival time, thus measuring the three-dimensional momentum of each electron. Since the electrons’ de Broglie wavelength of ∼0.1 nm is much below the smallest structure size of the sample, their trajectories are well described within the raytracing limit.^26^ The recorded UPEM images thus reflect their impact positions in the sample plane.

The antenna is excited by NIR pump pulses, linearly polarized perpendicular to the antenna slits under an angle of incidence of θ_*p*_ = 58°. Finite-difference-time-domain (FDTD) simulations predict enhancements of the transverse-field components of up to 20. The strongly enhanced field across the complete width of the slits allows for efficient electron–light interaction within the entire transparent region, resulting in a high interaction cross section.

For a qualitative understanding of the near-field electron coupling, we extend the approach introduced in ref (1) to a three-dimensional electron wavepacket (Supporting Information Section 1). We assume a wave function ψ(**r**, *t*) = *g*(**r** – **v**_0_*t*, *t*) exp(*i*(**k**_0_**r** – ω_0_*t*)) with wavevector **k**_0_ = (0,0,*k*_0_), angular frequency ω_0_, and envelope *g*(**r** – **v**_0_*t*, *t*), initially propagating in the *z*-direction at the group velocity **v**_0_ = (0,0,*v*_0_). Using the minimal coupling Hamiltonian *Ĥ*_*m*_ in the Coulomb gauge and neglecting the far-field components of the vector potential, the time-dependent Schrödinger equation reduces to
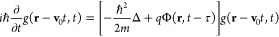
1Here, ℏ is the reduced Planck constant, *q* and *m* are the charge and mass of the free electron, and Φ(**r**, *t* – τ) is the pump induced scalar near-field potential. The time delay τ accounts for the delay between Φ and the arrival of the electron wavepacket in the sample plane. We neglect the dispersive term −ℏ^2^Δ*g*/(2*m*) during the few-fs interaction. The solution of eq 1 is then given as *g*(**r** – **v**_0_*t*_1_, *t*_1_) = *g*(**r** – **v**_0_*t*_0_, *t*_0_) exp (*i*Δφ(**r**, τ)), with the phase-modulation
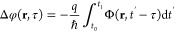
2where *t*_0_ and *t*_1_ define appropriately chosen times directly prior and after the interaction process. The phase modulation Δφ(**r**, τ) results in the emergence of a three-dimensional diffraction pattern in the probability distribution of the electron wavepacket in momentum space, *Ĩ*_*d*_(**k**, *t*_1_) = |*g̃*(**k** – **k**_0_, *t*_1_)|^2^, with **k** = (*k*_*x*_, *k*_*y*_, *k*_*z*_), while the charge density distribution in the spatial domain *I*_*d*_(**r**, *t*_1_) = |*g*(**r** – **v**_0_*t*_1_, *t*_1_) |^2^ remains unchanged by the near-field interaction. The envelope in momentum space *g̃*(**k**, *t*) can be obtained from *g*(**r** – **v**_0_*t*, *t*) by Fourier transform. Since *Ĩ*_*d*_(**k**, *t*_1_) = *Ĩ*_*d*_(**k**) remains unchanged upon propagation, the momentum distribution *Ĩ*_*d*_(**k**) measured by the detector directly reflects the momentum transfer between the optical near-field potential Φ and the electron.

Figure 1c,d shows the effect of the phase-modulation (eq 2) and the subsequent dispersion due to free-space propagation on the charge density distribution of a Gaussian-shaped 100 eV incident electron wavepacket (Figure 1c, left). Its pulse duration of 20 fs corresponds to a spatial extent of 120 nm. Here, the monochromatic near-field potential Φ(**r**, *t*) = Φ_0_(*y*, *z*) cos(*ω*_*p*_*t*) approximates the potential distribution around the antenna (Figure 1c, color-coded). The near-field interaction at delay τ = 0 results in a characteristic electron diffraction pattern in the momentum distribution *Ĩ*_*d*_(**k**) along both the longitudinal and transverse momentum directions. Distinct peaks along *k*_*z*_, spaced by the wavevector mismatch Δ*k* = ω_*p*_/*v*_0_ ≈ 0.17 nm^–1^, arise. These correspond to sidebands in the kinetic energy distribution spaced by the photon energy, as observed in PINEM experiments with swift electrons.^1,3,4^ Additional peaks appear along *k*_*y*_ at momenta ± Δ*k*_*y*_ ≈ 0.5 nm^–1^, inversely proportional to the transverse spatial near-field localization length.^19^ In real space (Figure 1c), the near-field coupling results in an undulatory modulation of the charge density along the transverse, *y*-direction. The wavepacket diffracts into a zero order peak, propagating along the *z*-direction, and sidepeaks with transverse momentum ± Δ*k*_*y*_. Since the average electron momentum *k*_0_ ≈ 50 nm^–1^ is small, the resulting diffraction angle θ_*y*_ ≈ Δ*k*_*y*_/*k*_0_ ≈ 0.5° is much larger than that for swift electrons. In addition, the emergence of photon sidebands along *k*_*z*_ will eventually result in a bunching of the charge density along the *z*-direction. This bunching appears roughly after *k*_0_/Δ*k* ≈ 300 cycles of the optical field and, thus, in the present simulations, after a few hundred femtoseconds. For swift electrons this time scale would be orders of magnitude higher due to the much larger wavevector *k*_0_ and the reduced wavevector mismatch Δ*k*.^4^

We start by experimentally probing the interaction of low-energy electrons with the optical near field of a Yagi-Uda antenna. The central kinetic energy of the electron probe pulses is set to *E*_0_ = 100 eV. The energy spread of the probe electrons is approximately Δ*E* = 3 eV. This results in a dispersive broadening of ∼20 fs due to free-space propagation from the tip to the sample (Supporting Information, Section 8). A typical UPEM image of the antenna, without an optical pump, is shown in Figure 2a, with a spatial resolution of ∼20 nm (Figure S10). Here the number of detected electrons *N*_*e*_(**r** = **r**_*d*_/*M*) is displayed as a function of their impact position in the detector plane, **r**_*d*_ = (*x*_*d*_, *y*_*d*_, *z*_*d*_), rescaled by the magnification *M*. Figure 2b shows the UPEM image in the presence of an optical pump pulse with an intensity of ∼14 GW/cm^2^, corresponding to an electric far-field amplitude of ∼0.3 V/nm. Optical pumping leads to blurring of the image, especially in the lower part of the antenna, where field enhancement is most pronounced. The difference image Δ*N*_*e*_ (pump on–pump off) reveals an increase in *N*_*e*_ around the slits and a decrease within them (Figure 2c). The total number of electrons changes by less than 3% in the presence of the pump, showing that optical pumping redistributes the electron distribution in the momentum space while leaving the overall transmission basically unchanged.

**Figure 2:**
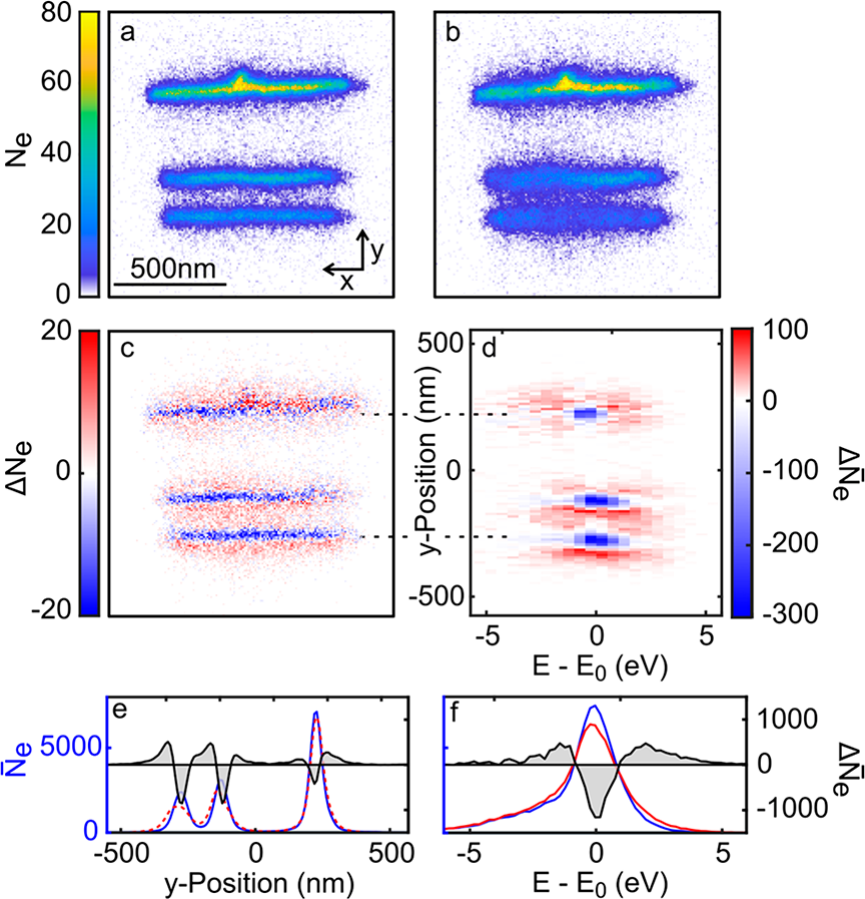
UPEM image of the Yagi-Uda antenna recorded with slow electrons with an energy *E*_0_ = 100 eV (a) without and (b) with optical pumping at 1900 nm. The data are taken during the temporal overlap of optical and electron pulses in the sample plane. (c) Difference images of (b)–(a), showing a decrease in the number of detected electrons *N*_*e*_ inside the antenna slits and an increase in the regions outside of the slits. (d) Difference in kinetic energy distribution of the transmitted electrons induced by optical pumping. Plotted is the pump-induced change in electron count Δ*N̅*_*e*_(*y*, *E* – *E*_0_) as a function of electron energy *E* and position *y*. The spectra are spatially integrated along the slit axis (*x*). Electrons impinging onto the slits with energy *E*_0_ are accelerated/decelerated by the near-field interaction and deflected along *y*. (e) Cross sections through the UPEM images in a (blue line, without pump) and b (red line, with pump), spatially integrated along the slit axis. The near-field deflection reduces the electron signal inside the slits and broadens the spatial distribution. The difference signal (gray area) emphasizes the deflection in the lower slits with strong field enhancement. (f) Spatially averaged kinetic energy spectra in the absence (blue line) and presence (red line) of the pump. The difference between both spectra (gray area) emphasizes the acceleration/deceleration of the electrons along their propagation direction.

The effect of optical pumping on the kinetic energy of the probe electrons is shown in Figure 2d. Here, we display the pump-induced change in the kinetic energy distributions of electrons Δ*N̅*_*e*_(*y*, *E* – *E*_0_) as a function of position *y*. The bar denotes the spatial integration of the spectra, here along the slit axis (*x*). The positions of the top and bottom slits of the antenna are marked by a dashed line as a guide to the eye. Inside the slits, i.e., in the regions of high transmission, the number of detected electrons is reduced, Δ*N̅*_*e*_ < 0, but the kinetic energy distribution is unaffected (blue spots). In the surrounding of the slits, the number of detected electrons increases, Δ*N̅*_*e*_ > 0, due to optical pumping (red spots). This increase in transmission is accompanied by distinct changes in the kinetic energy distribution. Electrons in the regions of enhanced transmission are transversally deflected in the *y*-direction and accelerated along *z* by the near-field interaction. This is evidenced by the two distinct lobes in the Δ*N̅*_*e*_(*y* ≈ 100 nm, *E* – *E*_0_) spectra. Similar correlations between near-field induced deflection and kinetic energy gain and loss are seen in the simulations shown in Figure 1d. Figure 2e shows cross sections through the UPEM images (a–c) along the antenna axis (*y*), integrated along the slits (*x*). Without an optical pump, the slits can be distinguished as three separate peaks (blue line). With an optical pump, the near-field interaction transversally deflects the probe electrons and therefore broadens the transmission *N̅*_*e*_(*y*) at each antenna slit (red dashed line). This broadening is clearly seen in the difference signal Δ*N̅*_*e*_(*y*) (gray area), emphasizing the dependence of the deflection on the local near-field enhancement, which is highest in the lowest slits (*y* = −250 nm) (Figure S13a). Figure 2f shows the spatially integrated kinetic energy distribution *N̅*_*e*_(*E* – *E*_0_) in the absence (blue line) and presence (red line) of the optical pump. The emergence of new kinetic energy contributions and the depletion around the central energy are evident in the difference spectrum Δ*N̅*_*e*_(*E* – *E*_0_) (gray area).

To investigate the dynamic aspects of the near-field interaction, we recorded UPEM images for different delay times τ. The experiments were performed on a similar Yagi-Uda antenna rotated by 180° around *z*. Since the antennas are optically excited under a finite angle, this rotation changes the field enhancements in the three slits (Figure S13b). Here, the pump intensity was set to ∼40 GW/cm^2^, corresponding to a maximum electric far-field amplitude of ∼0.5 V/nm.

The resulting UPEM images (Figure S8) are similar to those presented in Figure 2. Figure 3a,b shows the delay dependent kinetic energy distribution *N̅*_*e*_(*E* – *E*_0_, τ) of the probe electrons with (a) and without (b) an optical pump. As in Figure 2f, the energy distributions are spatially averaged over the entire UPEM image. Pump-induced changes in the spectrum are seen only in a narrow temporal window of ∼50 fs around time zero. The difference signals (b)–(a) in Figure 3c show this more clearly. Figure 3d,e displays cross sections of the delay-dependent electron distribution *N̅*_*e*_(*y*, τ), integrated along *x*. In the absence of the optical pump (d), the data show only a minor, slow drift of the sample position. During temporal overlap with the optical pump, some of the probe electrons are transversally deflected, as seen by the broadening and the decrease in amplitude of *N̅*_*e*_(*y*, τ = 0). This transient transverse deflection is better evidenced in the difference signal (e)–(d), displayed in Figure 3f. In contrast to the results shown in Figure 2, it is reduced for the two closer lying slits, revealing a larger field enhancement at the lowest slit.

**Figure 3:**
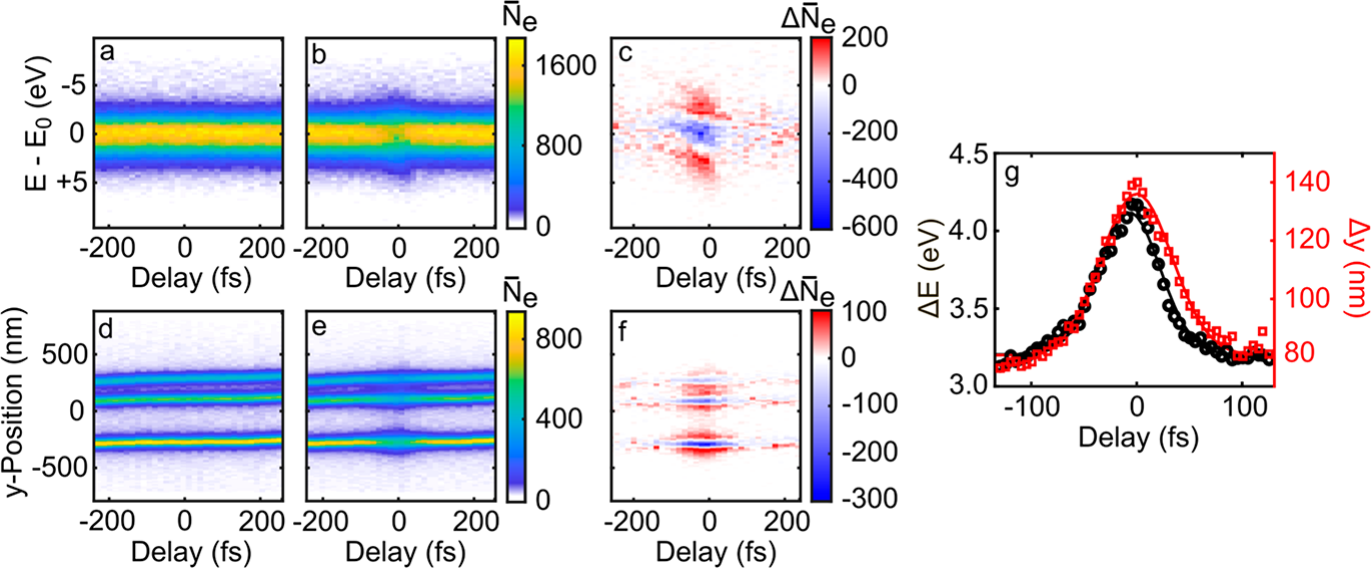
(a,b) Kinetic energy distribution, spatially averaged over the area of a Yagi-Uda antenna, as a function of the time delay between the optical pump and the electron probe (a) without and (b) with optical pumping. The electron-near-field interaction is restricted to ∼50 fs around time zero. The spectra are centered around *E*_0_ = 80 eV. (c) Difference signal (b)–(a), showing acceleration/deceleration of the probe electrons during their overlap with the optical pulse in the antenna plane. (d,e) Spatially and temporally resolved UPEM images of the antenna, integrated along the slit axis, in the absence (d) and presence (e) of the pump. (f) Difference signal (e)–(d), showing the transient, transverse deflection of the electrons around zero delay. (g) Width (fwhm) of the time-dependent kinetic energy distribution in (b) (black circles) and of the cross section along the *y*-direction of the upper split in (e) (red squares). Gaussian fits through the data have widths of 69 fs (black solid line) and 80 fs (red solid line), respectively. These widths are substantially longer than the initial duration of the electron pulse (20 fs) and that of the optical pump (20 fs).

To get quantitative insight into the electron near-field interaction, we analyzed the widths, Δ*E*(τ) (black circles), of the kinetic energy spectra *N̅*_*e*_(*E* – *E*_0_, τ), and Δ*y*(τ) (red squares), of the cross sections of the UPEM images *N̅*_*e*_(*y*, τ), taken for the slit at *y* ∼ −400 nm (Figure 3g). The energy distribution broadens from 3.2 eV at large delays to 4.2 eV around zero delay while the spatial width Δ*y*(τ) increases from 80 to 140 nm. The energetic broadening displays Gaussian-like dynamics with a full width at half-maximum (fwhm) of 70 fs, which is slightly longer than the duration of both the optical pump and the initial duration of the electron probe pulses. The transverse displacement follows the same dynamics at negative delays but persists somewhat longer (80 fs fwhm). The deflection corresponds to an increase in the transverse momentum of the electron wavepacket of Δ*k*_*y*_ ≈ 0.2 nm^–1^ while the energetic broadening can be translated into a change in the longitudinal momentum of Δ*k*_*z*_ ≈ 0.15 nm^–1^, in agreement with numerical simulations (Figure S2). This ultrafast response is very different from UPEM dynamics observed before for the interaction between probe electrons and free charges that were photoemitted from the sample.^22,23^ It is a distinct signature for a coherent interaction between the slow electrons and optical near fields in the sample plane, observed here for the first time.

In contrast to PINEM measurements with swift electrons,^2,4,8,34^ our experiments do not show distinct photon sidebands as a consequence of the coherent electron near-field interaction, but rather a broadening of the initial kinetic energy distribution. This difference results from the finite energetic width of our electrons of roughly 3 eV, substantially wider than the photon energy of the NIR photons. The coherent PINEM sidebands, therefore, are washed out (Figure S2a).^35−38^ To get more insight into the dynamics, the experiment in Figure 3 is repeated with a finer delay step size and an increased integration time. The differential kinetic energy spectra, Δ*N̅*_*e*_(τ, *E* – *E*_0_) in Figure 4a, again show a depletion around *E*_0_, similar to that in Figure 3c. Now, a transient shift in the kinetic energy distribution toward higher energies with increasing delay τ is noticeable, with a slope of roughly 0.025 eV/fs (black line).

**Figure 4:**
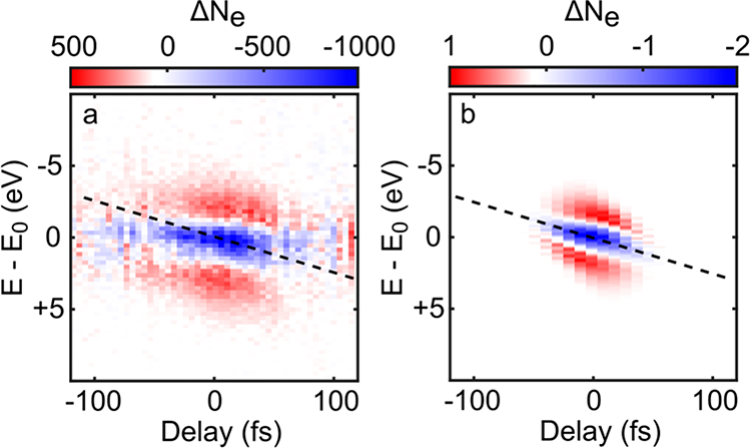
(a) Pump-induced change in the energy distribution Δ*N̅*_*e*_(τ, *E* – *E*_0_) of a probe electron pulse centered at *E*_0_ = 80 eV. The dashed line emphasizes the temporally asymmetric change in the spectrum. (b) Simulated change in spectrum, Δ*N̅*_*e*_(τ, *E* – *E*_0_), for a chirped electron pulse interacting with a near-field potential of 20 fs duration.

The same effect can be seen in delay-dependent simulations if we introduce a finite chirp of the electron wavepacket (Supporting Information Section 8). The simulated, time-varying energy distribution Δ*N̅*_*e*_(τ, *E* – *E*_0_), shown in Figure 4b, reproduces the slope seen in the experiment. In both cases, these transient energy shifts are a consequence of a finite chirp of the electron wavepacket. For τ < 0, the optical pump arrives after the center of the electron wavepacket and only the slower electrons couple to the near field. For τ > 0, in contrast, the near field interacts with the faster electrons, which arrive earlier. Thus, the negative peak in Δ*N̅*_*e*_, reflecting the near-field induced energetic broadening of the electron distribution, shifts from lower to higher energies with increasing τ. This temporal shift in energy thus provides a quantitative measure for the deduced electron chirp of 0.025 eV/fs.

FDTD simulations (Supporting Information Section 7) predict lifetimes of the optically excited symmetric eigenmode of the antenna array of ∼5 fs, which are too short to significantly affect the experimental dynamics. They also show, for the *y*-component of the field, spatially homogeneous field enhancements on the order of 10 inside the slits. This nicely accounts for the spatial dependence of the observed near-field electron deflection. They also suggest that the difference in dynamics seen in Figure 3g can be assigned to different near-field couplings for the longitudinal and transverse components.

This proof-of-principle demonstration of the interaction between localized near fields and low-energy electron pulses extends PINEM to low-energy, point projection microscopy, with a few tens of fs time resolution, for the first time. The deflection and acceleration dynamics in Figure 3c,f and Figure 4a are directly induced by the coherent coupling between the probe electron and the localized optical near field of the antenna. Hence, they are restricted to the temporal overlap between the electron probe and the optical pump. Probing such short-range, local optical near-field interactions goes beyond earlier experiments that have investigated long-range, incoherent Coulomb interactions between photoinduced charge carriers and probe electrons in ultrafast point-projection electron microscopy.^22,23^ Since the transverse deflection of low-energy electrons is large, this provides the time dynamics of all vector components of the local optical near field.

Building on this, a variety of future experiments are conceivable. A decrease in the kinetic energy spread of the probe electrons or an increase of the photon energy of the optical pump allows for resolving sidebands separated by the photon energy in the electron spectra. This brings the vast capabilities of PINEM to probe optical fields^40^ and to shape electron wave functions,^7^ to ultrafast low-energy electron microscopy. Photon induced near-field electron microscopy with swift electrons is currently undergoing rapid experimental development and is finding a broad range of new applications, for example, for creating coherent electron optical elements,^20^ generating new ways of holographic imaging,^41^ modifying the optical emission characteristics of solids,^42^ or probing the quantum properties of confined light fields.^43^ Our results suggests such work can now be extended to slow electron microscopy with much enhanced coupling to single confined modes. This will potentially catalyze the development of new types of sensors or novel quantum computing schemes.^7,44^ A reduction of the electron pulse duration into the subcycle regime brings streaking by optical near fields well within reach.^13,39^ This would allow for a complete spatiotemporal mapping of local optical fields at nanostructures and is the key to a controlled spatial and temporal shaping of low-energy electron pulses. Lastly, the use of low-energy electrons promises the application of ultrafast electron microscopy techniques to sensitive organic and biological nanomaterials.

## Data Availability

The data that support the findings of this study are available from the corresponding author upon reasonable request.

## Supporting Information Available

Theoretical model, simulation details, coupling strength estimation, laser system details, photoemission characteristics, sample preparation, detector system, data handling, spatial resolution, FDTD simulations, wavepacket chirp, phase matching (PDF)
