# Supplementary material for: Ultrafast Coupling of Optical Near Fields to Low-Energy Electrons Probed in a Point-Projection Microscope
Source: Nano Lett. 2023 Jun 6;23(12):5528–34. doi: 10.1021/acs.nanolett.3c00738 (PMC10311584; doi:10.1021/acs.nanolett.3c00738)
Supplement: Supplementary file 1 — nl3c00738_si_001.pdf [file nl3c00738_si_001.pdf]

Supporting Information

Ultrafast coupling of optical near fields to low energy electrons probed in a point-projection microscope

Andreas Wöste[1], Germann Hergert[1], Thomas Quenzel[1], Martin Silies[1], Dong Wang[2], Petra Groß[1], Christoph Lienau[1*]

1. Institut für Physik und Center for Nanoscale Dynamics (CeNaD), Carl von Ossietzky Universität, 26129 Oldenburg, Germany

2. Institut für Werkstofftechnik und Institut für Mikro- und Nanotechnologien, TU Ilmenau, 98693 Ilmenau, Germany

Author e-mail address: christoph.lienau@uol.de

1. Theoretical modelling

We consider the interaction of an ultrashort, slow electron wave packet with a spatially localized strong optical near-field created in the vicinity of a nanometer-sized structure. We assume that the electron wave packet is described by a wavefunction $\psi(\mathbf{r},t)$, where t is the time and $\mathbf{r} = x\mathbf{e}_x + y\mathbf{e}_y + z\mathbf{e}_z$ defines a Cartesian coordinate system in space. In order to get insight into the spatio-temporal evolution of the electron wavepacket dynamics, we seek solutions of the time dependent Schrödinger Equation (TDSE), following the approach introduced by Park et al.[1],

$$i\hbar \frac{\partial}{\partial t}\psi(\mathbf{r},t) = \hat{H}_m \psi(\mathbf{r},t)\,. \tag{1}$$

We use the minimal coupling Hamiltonian for non-relativistic free electrons

$$\hat{H}_m = \frac{1}{2m}\left(\hat{\mathbf{p}} - q\mathbf{A}\right)^2 + q\Phi = \frac{1}{2m}\left(-i\hbar\nabla - q\mathbf{A}\right)^2 + q\Phi\,. \tag{2}$$

Here, $\hbar$ is the reduced Planck constant, $\hat{\mathbf{p}} = -i\hbar\nabla$ the momentum operator and $\mathbf{A}$ and Φ are the classical vector and scalar potential, respectively. The constants q and m denote charge and mass of the free electron. We neglect transverse components of $\mathbf{A}$ since propagating electromagnetic fields do not couple to the electron due to a finite wavevector mismatch[1]. Additionally, we use the Coulomb gauge, $\nabla \cdot \mathbf{A} = 0$ which sets longitudinal components of $\mathbf{A}$ to zero. Consequently, only the optical near fields in close vicinity of the sample are considered for the electron-light interaction and the Hamiltonian reduces to

$$\hat{H}_m = \frac{\hat{\mathbf{p}}^2}{2m} + q\Phi(\mathbf{r}, t-\tau) = -\frac{\hbar^2}{2m}\Delta + q\Phi(\mathbf{r}, t-\tau), \tag{3}$$

where we introduced a time delay τ between the creation of the optical near-field and the arrival of the electron wave packet in the sample plane. This time delay is set by the pump-probe delay in our optical pump – electron probe experiments. We further separate the wavefunction into a product of an envelope function g, moving at the group velocity $\mathbf{v}_0$ of the incident electron wave packet and a carrier wave $\psi_0(\mathbf{r},t) = \exp(i\mathbf{k}_0\mathbf{r} - i\omega_0 t)$ with the average wavevector $\mathbf{k}_0$ and angular frequency ω_0,

$$\psi(\mathbf{r},t) = g(\mathbf{r} - \mathbf{v}_0 t, t)\,\psi_0(\mathbf{r},t) \tag{4}$$

Following Ref. [1], we introduce the moving frame $\mathbf{r}' = \mathbf{r} - \mathbf{v}_0 t$ and substitute Eqs. (3) and (4) into Eq. (1). This gives

$$
\begin{aligned}
& i\hbar \frac{\partial}{\partial t}\psi(\mathbf{r},t) = \hat{H}_m \psi(\mathbf{r},t) \\
& i\hbar \frac{\partial}{\partial t}\left(g(\mathbf{r}-\mathbf{v}_0 t,t)\,\psi_0(\mathbf{r},t)\right) = \hat{H}_m\left(g(\mathbf{r}-\mathbf{v}_0 t,t)\,\psi_0(\mathbf{r},t)\right) \\
& i\hbar\left(\dot{g}\,\psi_0 - \mathbf{v}_0\nabla g\psi_0 - i\omega_0 g\psi_0\right) = -\frac{\hbar^2}{2m}\left(\Delta g\,\psi_0 + 2\nabla g\nabla\,\psi_0 + g\,\Delta\psi_0\right) + q\Phi g\,\psi_0 \\
& \dot{g} - \mathbf{v}_0\nabla g - i\omega_0 g = \frac{i\hbar}{2m}\Delta g - \frac{\hbar\mathbf{k}_0}{m}\nabla g - i\frac{\hbar\left|\mathbf{k}_0\right|^2}{2m} g - i\frac{q}{\hbar}\Phi g \\
& i\hbar\dot{g} = -\frac{\hbar^2}{2m}\Delta g \; + q\Phi g
\end{aligned}
\tag{5}
$$

Here, $\dot{g} = \partial g\left(\mathbf{r}',t\right)/\partial t\big|_{\mathbf{r}'=\mathbf{r}-\mathbf{v}_0 t}$, is the time derivative in the moving frame. The spatial derivatives are identical in the moving and in the rest frame. Since the speed of the electron wavepacket is ~ 0.02 c_0 (c_0: speed of light in vacuum) for ~ 100-eV electrons and the spatial extent of the near field is on the order of 20 nm, the interaction time is restricted to ~ 5 fs. During this time, the effect of the near-field acceleration on the spatial shape of the electron density $\left|\psi\left(\mathbf{r},t\right)\right|^2$ is small. Therefore, in order to solve Eq.(5), we make the approximation that the dispersive term $-\hbar^2\Delta g/\left(2m\right)$ can be neglected during the interaction of the electron wave packet with the near field. The solution of Eq. (5) within this approximation is given by integration with respect to time from time t_0, directly prior to the interaction, to time t_1 directly after the interaction. The interaction with the near field then introduces a modulation of the local phase of the electron wavepacket $\Delta\varphi\left(\mathbf{r},\tau\right)$

$$
\begin{aligned}
g(\mathbf{r}-\mathbf{v}_0 t_1,t_1) &= g(\mathbf{r}-\mathbf{v}_0 t_0,t_0)\exp\left(-i\frac{q}{\hbar}\int_{t_0}^{t_1}\Phi(\mathbf{r},t'-\tau)dt'\right) \\
&= g(\mathbf{r}-\mathbf{v}_0 t_0,t_0)\exp\left(i\Delta\varphi\left(\mathbf{r},\tau\right)\right)
\end{aligned}
\tag{6}
$$

Evidently, the phase modulation does not depend on the precise choice of t_0 and t_1 as long as the near-field interaction is fully covered. This phase modulation translates into a change of the three-dimensional momentum distribution

$$\tilde{I}_d\left(\mathbf{k},t_1\right)=\left|\tilde{g}\left(\mathbf{k}-\mathbf{k}_0,t_1\right)\right|^2 \tag{7}$$

of the electron wavepacket. Here, $\tilde{g}\left(\mathbf{k},t\right)$ is the three-dimensional Fourier transform of the envelope $g\left(\mathbf{r}',t\right)$ in the moving frame, centered around $\mathbf{k}=0$. After the interaction with the optical near field $\left(t>t_1\right)$, the scalar potential Φ can be set to zero and changes in g solely arise from the dispersion term. Consequently Eq. (5) reduces to

$$i\hbar\dot{g}(\mathbf{r}-\mathbf{v}_0 t,t)=-\frac{\hbar^2}{2m}\Delta g(\mathbf{r}-\mathbf{v}_0 t,t)\,. \tag{8}$$

In momentum space, this corresponds to

$$\dot{\tilde{g}}(\mathbf{k},t)=-i\frac{\hbar\mathbf{k}^2}{2m}\tilde{g}(\mathbf{k},t)\,. \tag{9}$$

Wave packet propagation thus results in a phase modulation of $\tilde{g}$

$$\tilde{g}(\mathbf{k},t)=\tilde{g}(\mathbf{k},t_1)\exp\left(-i\frac{\hbar}{2m}\mathbf{k}^2\left(t-t_1\right)\right). \tag{10}$$

Since the momentum distribution $\left|\tilde{g}\right|^2$ does not change with propagation time, our experiment directly measures, via Eq. (7), the electron distribution in momentum space at time t_1, directly after the near-field interaction, centered around $\mathbf{k}_0$. While it is known that the separation of the

time-integration of Eq. (5) into two distinct steps (near-field interaction vs. beam dispersion) is well justified for swift electrons, it is less obvious whether this is also a good approximation for slow electrons. Direct numerical evaluation of Eq. (5) suggests that this approximation does not visibly change the resulting electron kinetic energy distributions[2].

The simulations shown in the main manuscript were performed by numerically solving Eq. (5) in the two spatial dimensions y and z. The x-direction was omitted, since the near-field components along the slit axis of the Yagi-Uda antenna are weak. Hence, the problem can be restricted to solving a two-dimensional Schrödinger equation. The associated potential $\Phi(y,z,t)$ is modelled by the superposition of the potential distribution of two dipoles, which oscillate along the y-direction at frequency ω_p and are separated by the distance $d = 46.5$ nm, at $z = 0$. Taking their dipole moment as p_0 and the vacuum permittivity ε_0, the resulting potential is given by:

$$\Phi_D(y,z)\cos\left(\omega_p t\right) = \frac{p_0}{4\pi\varepsilon_0}\left[\frac{y+d/2}{\sqrt{z^2+\left(y+d/2\right)^2}^3} + \frac{y-d/2}{\sqrt{z^2+\left(y-d/2\right)^2}^3}\right]\cos\left(\omega_p t\right) \qquad (11)$$

Here $\Phi_D(y,z)$ describes the spatial profile of the potential of the two dipoles. To account for the finite sample thickness in the experiment, the potential distribution is convoluted with a two-dimensional Gaussian function $G_r\left(y,z\right)$, such that $\Phi(y,z,t)$ is given by $\Phi(y,z,t) = \Phi_D(y,z)\cos\left(\omega_p t\right) \otimes G_r\left(y,z\right) = \Phi_0\left(y,z\right)\cos\left(\omega_p t\right)$. Thus, $\Phi_0 = \Phi_D \otimes G_r$ is the spatial profile of the near field potential. The full width at half maximum (FWHM) of $G_r\left(y,z\right)$ is set to 13 nm in both dimensions, matching the sample thickness along the propagation direction z. The

potential $\Phi(y,z,0)=\Phi_0\left(y,z\right)$ used in the simulations shown in Fig. 1c,d is displayed in Fig. S1a. The resulting electric field components E_y and E_z are added together with the magnitude $E_{mag}=\sqrt{E_z^2+E_y^2}$ in Figs. S1b-d. In the simulations, the maximum field amplitudes reach 2 V/nm. In experiment, this corresponds to an incident far-field amplitude of 0.1 – 0.2 V/nm, reasonably close to our experimental values when considering the typical field enhancements of 10-20 in Fig. S11. The spatial charge distribution and the momentum distribution of the electron wavepacket, prior to the interaction with the optical potential, is shown in Fig. S1e and S1f, respectively. Since, the electron wavepacket is localized to ~20 nm in transverse, y-direction, the dipole approximation in Eq. (11) is sufficient to describe the electric field seen by the electron.

In the experiments, however, the width of the kinetic energy distribution of the electrons of roughly 3 eV is substantially wider. At a wavelength of 1900 nm (0.65 eV), the absorption of at least 8 photons is needed to overcome the work function of gold and to release a photoelectron from the tip. Such high order multiphoton emission processes require local electric field amplitudes at the tip apex of the order of 5-10 V/nm. Under these emission conditions, the ponderomotive acceleration of the electrons in the near field of the tip is substantial, resulting in a few eV broadening of their kinetic energy distribution. Also, the pump-induced heating of the electron gas by the pump pulses contributes to the energetic broadening.[3–6] Much narrower photoemission spectra can be obtained by using visible or near-UV excitation pulses and/or Schottky-type field emitters with reduced work functions.[7] The impact of the energetic width on the result of the electron near-field interaction is shown in Fig. S2a.

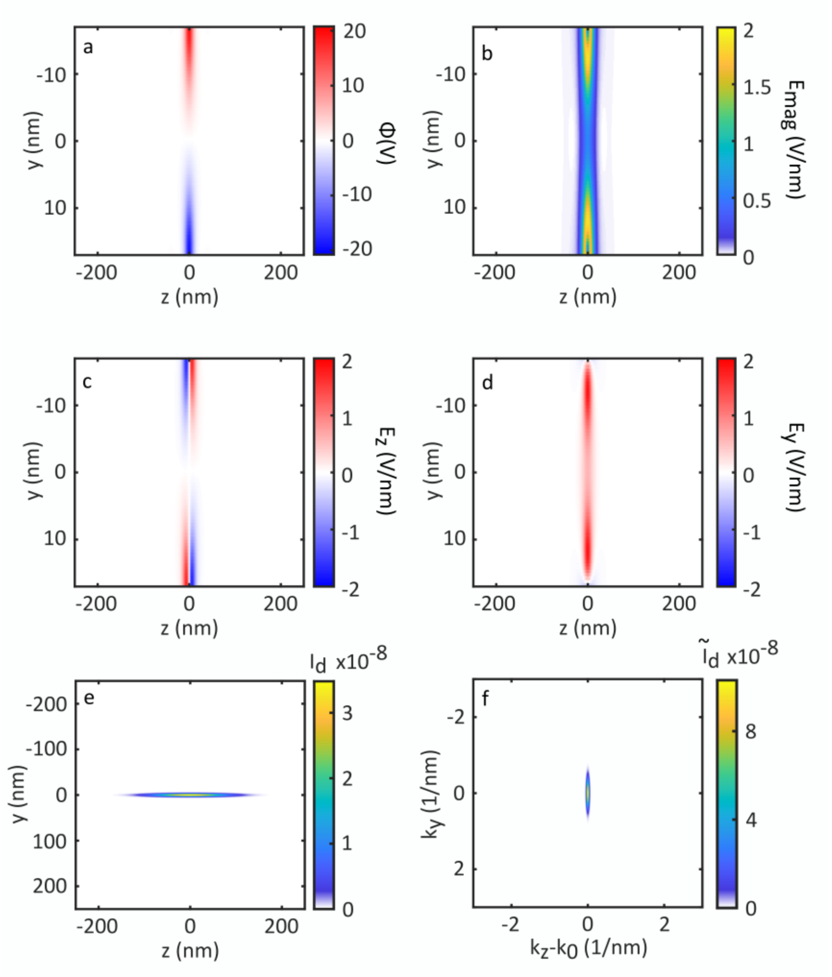

Figure S1. (a) Potential and (b-d) electric fields for t = 0. (e) Initial charge and (f) momentum distribution of the electron wavepacket that is shown in Fig. 1c,d in the manuscript.

Here, the appearance of photon sidebands in the spectrum of a 20-fs, 100-eV electron wavepacket, interacting with an antenna near-field as displayed in Fig. S1, is analyzed. For a narrow energy distribution ($\Delta E = 0.2$ eV), sidebands at the photon energy $\hbar\omega_p$ are clearly resolved (top, red curve). These sidebands are washed out for a broader distribution with $\Delta E = 2$ eV (bottom, red curve). The near-field deflection of the electron distribution, expressed in terms of its transverse momentum distribution along k_y, does not depend strongly on ΔE, shown in Fig. S2b. In both cases the initially Gaussian distribution (blue area) broadens symmetrically

towards higher and lower values of k_y. This change in the transverse momentum distribution leads to a symmetric deflection of the electron wavepacket in positive and negative y-direction. When choosing a maximum field strength of ~2 V/nm in the simulations, the change in kinetic energy and the transverse momentum are on the same order as those seen in the experimental data. Electron deflection and change in the kinetic energy distribution are therefore explained by an interaction between the optical antenna near-field and the low energy electron wavepacket similar to the interaction seen in photon-induced near-field electron microscopy (PINEM) using swift electrons.

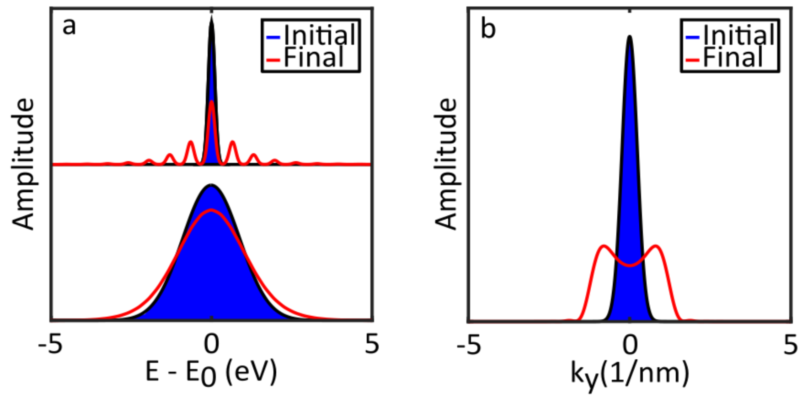

Figure S2. (a) Simulated kinetic energy spectra of a 20-fs, 100-eV electron pulses prior (blue) and after (red) the interaction with the transient optical near-field of a Yagi-Uda antenna. Photon sidebands are resolved for a narrow incident spectrum, ($\Delta E = 0.2$ eV, (a) top), yet washed out for broader spectra ($\Delta E = 2$ eV, (a) bottom) that are typical for multiphoton photoemission from gold tapers. (b) Transverse momentum distribution before (blue) and after (red) the near-field interaction, showing the deflection of the passing electrons.

2. Estimate of the coupling constant

In principle, Eqs. (6) and (7) are sufficient to describe the effect of the near-field coupling on the probe electrons. Often, however, Eq. (6) is expressed in terms of a dimensionless coupling

constant, which defines the overall strength of the interaction, integrated along the electrons trajectory[1,2,8-10]. For this, we consider the phase modulation of the envelope g in the moving frame. After substituting $\mathbf{r}'=\mathbf{r}-\mathbf{v}_0 t$ Eq. (6) reads

$$g(\mathbf{r}',t_1)=g(\mathbf{r}',t_0)\exp\left(i\Delta\varphi\left(\mathbf{r},\tau\right)\right), \tag{12}$$

with

$$\Delta\varphi(\mathbf{r},\tau)=-\frac{q}{\hbar}\int_{t_0}^{t_1}\Phi(\mathbf{r}'+\mathbf{v}_0 t',t'-\tau)dt' . \tag{13}$$

We substitute the variables within the integral part by $\mathbf{r}''=\mathbf{r}'+\mathbf{v}_0 t'$, i.e., we switch from an integral over the time domain to a line integral along the trajectory of the electron. Importantly, the position $\mathbf{r}''$ now is in the laboratory frame, not in the moving frame. With

$$\begin{aligned} t' &= \frac{\mathbf{v}_0}{\left|\mathbf{v}_0\right|^2}\left(\mathbf{r}''-\mathbf{r}'\right) \\ dt' &= \frac{\mathbf{v}_0}{\left|\mathbf{v}_0\right|^2}d\mathbf{r}'' \end{aligned} \tag{14}$$

the phase modulation can be expressed as

$$\Delta\varphi=-\frac{q}{\hbar\left|\mathbf{v}_0\right|^2}\int_{\mathbf{r}''(t_0)}^{\mathbf{r}''(t_1)}\Phi\left(\mathbf{r}'',\frac{\mathbf{v}_0}{\left|\mathbf{v}_0\right|^2}\left(\mathbf{r}''-\mathbf{r}'\right)-\tau\right)\mathbf{v}_0 d\mathbf{r}'' . \tag{15}$$

This readily allows us to calculate the influence of the near-field interaction on the electron wave packet, given that we know the shape and the time evolution of the near-field potential Φ. We

express the near-field potential Φ as the superposition of monochromatic waves at frequency ω with the space- and frequency dependent amplitudes $\Phi_G(\mathbf{r},\omega)$ and phase $\varphi(\omega)$

$$\Phi(\mathbf{r},t)=\int \Phi_G(\mathbf{r},\omega)\cos\left(\omega t+\varphi(\omega)\right)d\omega\,. \tag{16}$$

Inserting Eq. (16) into Eq. (15) gives

$$\Delta\varphi=-\frac{q}{\hbar|\mathbf{v}_0|^2}\int_{\mathbf{r}''(t_0)}^{\mathbf{r}''(t_1)}\int \Phi_G(\mathbf{r}'',\omega)\cos\left(\omega\frac{\mathbf{v}_0}{|\mathbf{v}_0|^2}(\mathbf{r}''-\mathbf{r}')-\omega\tau+\varphi(\omega)\right)d\omega\mathbf{v}_0 d\mathbf{r}''\,. \tag{17}$$

Introducing the momentum mismatch $\Delta\mathbf{k}_0=\omega\frac{\mathbf{v}_0}{|\mathbf{v}_0|^2}$, Eq. (17) reads

$$\Delta\varphi=-\frac{q}{\hbar|\mathbf{v}_0|^2}\int_{\mathbf{r}''(t_0)}^{\mathbf{r}''(t_1)}\int \Phi_G(\mathbf{r}'',\omega)\cos\left(\Delta\mathbf{k}_0\mathbf{r}''-\Delta\mathbf{k}_0\mathbf{r}'-\omega\tau+\varphi(\omega)\right)d\omega\mathbf{v}_0 d\mathbf{r}''\,. \tag{18}$$

We can express the cosine term by taking the real part of its exponential representation

$$\Delta\varphi=-\frac{q}{\hbar|\mathbf{v}_0|^2}\int_{\mathbf{r}''(t_0)}^{\mathbf{r}''(t_1)}\int \Phi_G(\mathbf{r}'',\omega)\Re\left\{\exp\left(i\Delta\mathbf{k}_0\mathbf{r}''\right)\exp\left(-i\Delta\mathbf{k}_0\mathbf{r}'-i\omega\tau+\varphi(\omega)\right)\right\}d\omega\mathbf{v}_0 d\mathbf{r}''\,. \tag{19}$$

Since the potential $\Phi_G(\mathbf{r}'',\omega)$ is real valued Eq. (19) can be reformulated as

$$\Delta\varphi=-\Re\left\{\int\left(\frac{q}{\hbar|\mathbf{v}_0|^2}\int_{\mathbf{r}''(t_0)}^{\mathbf{r}''(t_1)}\Phi_G(\mathbf{r}'',\omega)\exp\left(i\Delta\mathbf{k}_0\mathbf{r}''\right)\mathbf{v}_0 d\mathbf{r}''\right)\exp\left(-i\Delta\mathbf{k}_0\mathbf{r}'-i\omega\tau+\varphi(\omega)\right)d\omega\right\}. \tag{20}$$

Defining the frequency-dependent coupling integral $I(\omega)$ as

$$I(\omega) = -\frac{q}{\hbar|\mathbf{v}_0|^2} \int_{\mathbf{r}''(t_0)}^{\mathbf{r}''(t_1)} \Phi_G(\mathbf{r}'', \omega) \exp(i\Delta\mathbf{k}_0\mathbf{r}'') \mathbf{v}_0 d\mathbf{r}'', \tag{21}$$

the near-field induced phase modulation can be expressed as

$$\Delta\varphi = \Re\left\{\int I(\omega) \exp(-i\Delta\mathbf{k}_0\mathbf{r}' - i\omega\tau + \varphi(\omega)) d\omega\right\}. \tag{22}$$

In the case of a monochromatic optical field $\omega = \omega_p$, and for $\varphi = 0$ and $\tau = 0$, Eq. (22) reduces to

$$\Delta\varphi(\mathbf{r}, 0) = \Re\left\{I(\omega_p) \exp(-i\Delta\mathbf{k}_0\mathbf{r}')\right\}. \tag{23}$$

Restricting the propagation direction of the electron to one dimension, the z-direction, the coupling integral reduces to

$$\begin{aligned} I(x,y)\Big|_{\mathbf{v}_0 = v_0\mathbf{e}_z} &= -\frac{q}{\hbar|\mathbf{v}_0|^2} \int_{\mathbf{r}''(t_0)}^{\mathbf{r}''(t_1)} \Phi_G(\mathbf{r}'', \omega_p) \exp(i\Delta\mathbf{k}_0\mathbf{r}'') \mathbf{v}_0 d\mathbf{r}'' \Bigg|_{\mathbf{v}_0 = v_0\mathbf{e}_z} \\ &= -\frac{q}{\hbar v_0} \int_{z''(t_0)}^{z''(t_1)} \Phi_G(x, y, z'', \omega_p) \exp(i\Delta k_z z'') dz'' \end{aligned}. \tag{24}$$

The coordinates in the last line of Eq. (24) are again x, y instead of x'', y'' since for $v_x = v_y = 0$ $x'' = x' = x$, and $y'' = y' = y$, by definition. In this representation the coupling integral provides the Fourier components of the near-field at the spatial frequency of the wavevector mismatch $\Delta k_z = \omega_p / v_0$ [1, 2]. At a given coordinate (x_i, y_i) the magnitude of the dimensionless value $|I(x_i, y_i)|$ equals $2|g_c|$, with the dimensionless coupling constant g_c as defined by Feist et al.[10].

We have calculated the spatially dependent coupling constant g_c for the field parameters shown in Fig. S1 (blue line in Fig. S3). Close to the edges of the slits, g_c can reach values of up to 20. Here, however, the charge density distribution (red line in Fig. S3) is small. The coupling constant decreases to zero towards the center of the slit, where the amplitude of the wavefunction is highest. When spatially averaging the coupling constant, weighted by the wavefunctions charge density distribution amplitude, along the y -direction, an average value of $g_c \approx 2$ is obtained. This value corresponds well to the energetic broadening of the wavepacket from 3 e V to 4.5 eV that is seen in our ultrafast point-projection electron microscope (UPEM) experiments.

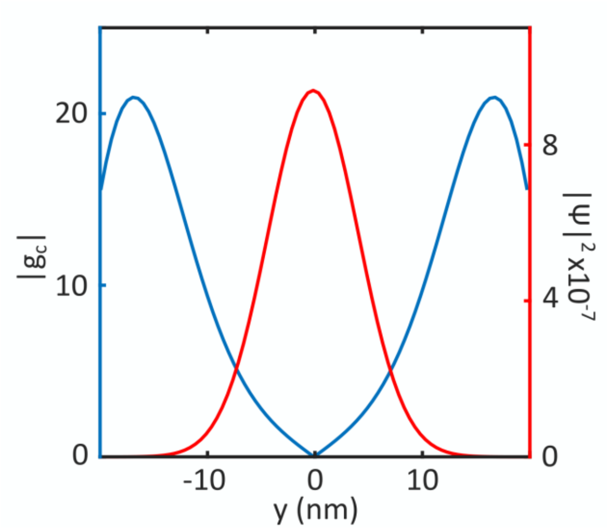

Figure S3. Variation of the coupling constant $g_c(y)$, as calculated for the near-field potential shown in Fig. S1a using Eq. (24)*, along the direction y perpendicular to the slit axis(blue line). The red line shows the spatial variation of the charge density distribution. A spatial average of the coupling constant, weighted by the charge distribution, gives a value of $g_c \approx 2$.*

3. Generation of few-cycle near-infrared laser pulses.

Few-cycle near infrared (NIR) laser pulses are generated in a home-built laser system, following a design reported in References [11] and [12]. The amplifier system is divided into a noncollinear optical parametric amplifier (NOPA) (bottom panel in Fig. S4), generating pulses in the visible and a difference frequency generation (DFG) stage (top panel in Fig. S4), creating the near-infrared pulses that are used to trigger photoemission from the tip and to excite the antenna. A commercial fiber laser (Amplitude Systèmes, Tangerine) is operated at 175 kHz repetition rate and provides 250-fs output pulses at a central wavelength around 1030 nm and with an average power of 35 W, providing a pulse energy of 200 µJ. A fraction of these seed pulses, with ~1.7 μJ pulse energy, is used to create white light (WL) by supercontinuum creation in a 4-mm long Yttrium-Aluminium-Garnet (YAG) crystal. The WL pulses (~10 nJ) are focused into a Beta Barium Borate (BBO) crystal with a thickness of 2 mm, cut at an angle of 22.3°. The BBO crystal is simultaneously pumped with pulses centered around 515 nm (~40 μJ), created by second harmonic generation (SHG) from a fraction of the seed pulses (~65 μJ) in a 1-mm thick BBO crystal, cut at 23.4°. In the NOPA, a part of the WL spectrum, around 670 nm, is amplified to a pulse energy of ~0.7 μJ by optical parametric amplification. The amplified pulses are collinearly combined with another fraction of the seed pulses (~3.7 μJ) and focused into a 1-mm thick BBO, cut at 23.4°. Here NIR pulses, with ~100-nJ pulse energy, are created by DFG. Compression of the NIR pulses is achieved by tuning the dispersion of the amplified WL pulses prior to the DFG.

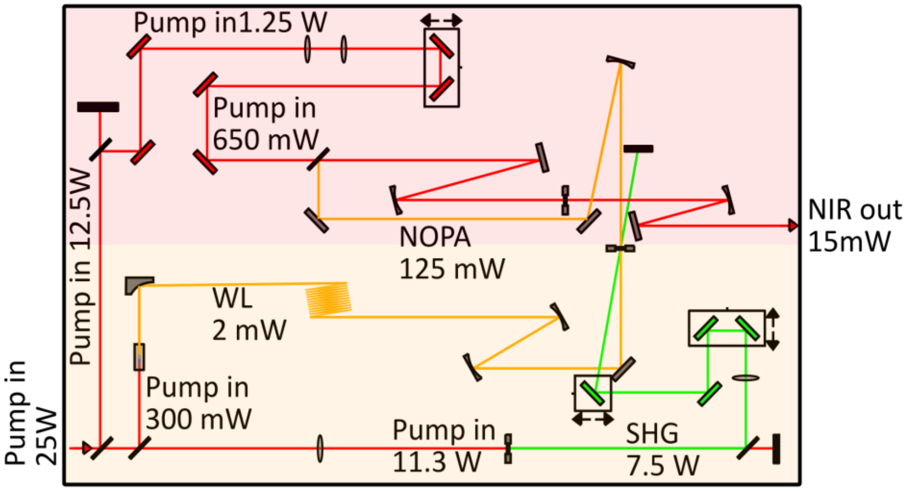

Figure. S4. Sketch of the amplifier system. Near infrared pulses (NIR) centered around 1900 nm are created by difference frequency generation (DFG) between seed pulses centered around 1030 nm and visible pulses centered around 670 nm. The DFG section is shown at the top (red) and the visible NOPA at the bottom (yellow).

The pulse duration of the NIR pulses is measured in a home built interferometric frequency-resolved autocorrelation (IFRAC) setup[13]. Here, the second harmonic (SH) spectrum, created within a 30 μm thin BBO, under collinear illumination with a phase-locked pair of NIR pulses with electric field $E(t)$ is measured as a function of the delay τ between the pulses. The resulting two-dimensional intensity distribution I_{IFRAC} as a function of the SH frequency $\omega = 2\pi c_0 / \lambda$ is given as [13, 14]:

$$I_{IFRAC}(\omega,\tau) \propto \left| \int_{-\infty}^{\infty} \left(E(t)+E(t-\tau)\right)^2 \exp(-i\omega t)\, dt \right|^2 . \qquad (25)$$

Here, we assume a quasi-instantaneous SH generation in the BBO crystal, generating a second harmonic field $E_{SH}(t,\tau) = \left(E(t)+E(t-\tau)\right)^2$. Using Fourier transform and filtering techniques, a frequency-resolved optical gating spectrum (FROG) spectrum $I_{FROG}(\omega,\tau)$ (plotted as a function

of the SH wavelength λ in Fig. S5a) can be isolated from the measured IFRAC signal[13] and be written as:

$$I_{FROG}(\omega,\tau) \propto \left| \int_{-\infty}^{\infty} E(t)E(t-\tau)e^{-i\omega t}dt \right|^2 . \quad (26)$$

From I_{FROG} the spectral and temporal structure of the optical pulse can be retrieved[15]. The retrieved signal $\tilde{I}_{FROG}$ is shown in Fig. S5b. The retrieved spectral intensity (black line) and phase (orange line) is shown in Fig. S5c, together with the spectrum measured by a spectrometer (A.P.E waveScan) (blue line). Experimental and simulated data show good agreement and indicate a group delay dispersion (GDD) of ~30 fs^2 and a third order dispersion (TOD) of -2000 fs^3. Fitting the retrieved temporal intensity distribution with a Gaussian function (Fig. S5d, orange line) yields a pulse duration of ~18 fs FWHM, slightly longer than the 15 fs bandwidth limit. The TOD mostly influences the amplitude of the trailing (or leading) side peak, seen around 25 fs in Fig. S5d.

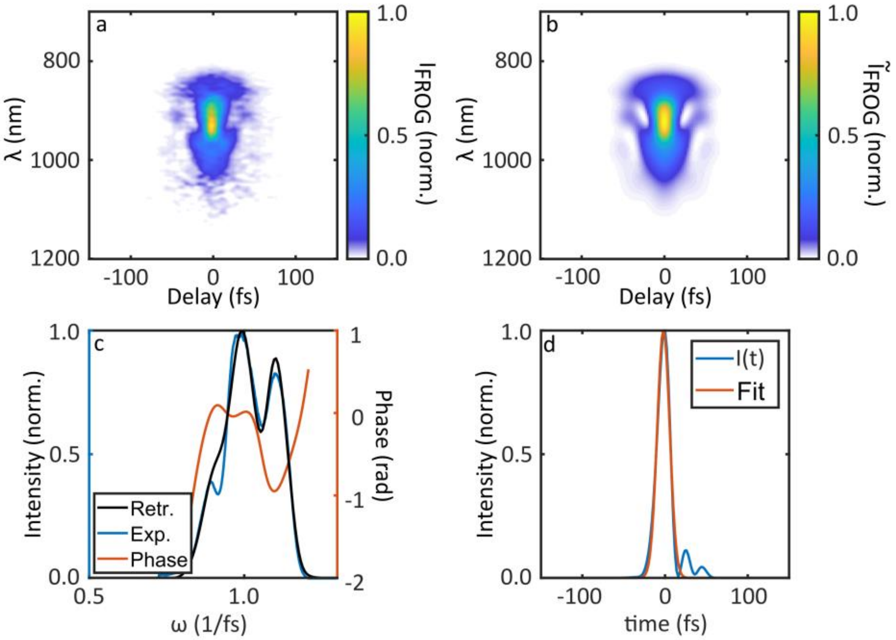

Figure. S5. (a) Frog spectrum I_{FROG} extracted from an IFRAC measurement of the NIR laser pulses. (b) Retrieved distribution $\tilde{I}_{FROG}$. (c) Spectrum of the NIR pulses measured with a spectrometer (blue line). Spectrum and phase as retrieved from the IFRAC measurement are shown by the black and orange line. (d) Retrieved intensity over time, revealing a pulse duration of around 20fs.

To confirm the time resolution of the optical pump – electron probe experiment at the position of the gold nanotaper, we record an interferometric electron cross-correlation trace (ECC)[16]. The ECC is obtained by detecting the photoelectron emission rate from the nanotaper, under illumination with a pair of counter-propagating NIR pulses with electric fields $E_1(t)$ and $E_2(t)=E_1(-t-\tau)$. The emission rate $C(\tau)$ is measured as a function of the time delay τ between the two pulses. The emission rate is assumed to scale nonlinearly with the total electric field strength at the tip apex $E_T(t,\tau)=E_1(t)+E_1(-t-\tau)$, such that the detected emission rate is given by:

$$C(\tau)\propto\int_{-\infty}^{\infty}\left[\theta(E_T)E_T\right]^{2N}dt\,. \qquad (27)$$

Here, N describes the order of the nonlinearity of the photoemission process and $\theta(x)$ denotes the Heaviside function. By introducing the Heaviside function we assume that photoelectron emission occurs only during the half cycles for which the local field points towards the tip center, driving the electron into the vaccum[5]. A measurement of the ECC is shown together with a fit following Eq. (27) in Fig. S6a. For the simulation, $E_1(t)$ was calculated from the measured NIR spectrum (Fig. S5c). The dispersion of the pulses (GDD and TOD) and the nonlinearity N of the emission process were adjusted to fit the measurement data. Good agreement with the

experiment was reached for N = 6, a GDD of 130 fs^2 and a TOD of -600 fs^3. The Intensity distribution $I(t) \propto E_1^2(t)$ obtained from the fit is shown in Fig. S6b. Compared with the results obtained from the IFRAC measurement, the value of the obtained GDD and TOD parameters changed slightly. This difference might result from the propagation of the plasmon over 80 µm from the grating coupler towards the apex of the gold tip (see section 4)[17]. The retrieved laser pulse duration at the apex is 21 fs, confirming that short pulse durations are reached at the apex of the taper by plasmonic nanofocusing. Based on the 6th order nonlinearity of the emission process, we estimate a minimum initial electron pulse duration at the apex of 21 fs/$\sqrt{6}$ = 9 fs.

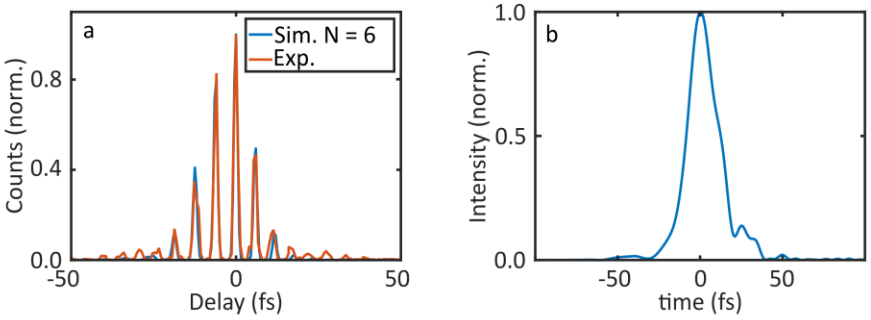

Figure S6. (a) Electron cross-correlation photoemission signal from the gold nanotaper using time delayed, counter propagating duplicates of the NIR laser pulses, measured (orange) and simulated (blue). (b) Intensity distribution of the incident laser, $I(t) \propto E_1^2(t)$, obtained from the fit indicating a laser pulse duration of 21 fs at the apex.

4. Gold tip and sample preparation

The gold tip, used as the photoemission source for the electron probe pulses, was electrochemically etched from a monocrystalline gold wire[17]. Scanning electron microscope (SEM) images of the taper and the apex are shown in Figs. S7a and S7b, respectively. They reveal a tip radius of less than 15 nm which allows us to reach high spatial resolution in the point-

projection microscopy experiments[18]. The use of a monocrystalline wire results in a particularly smooth taper surface, enabling efficient nanofocusing of plasmons that are launched at the grating coupler[13]. The distance of 80 µm between grating and apex is substantially larger than the 30 µm spot size of the NIR pulses that are focused on the grating coupler (see Fig. 1a). This ensures that neither the tip apex nor the sample are strongly excited by the illumination of the grating coupler.

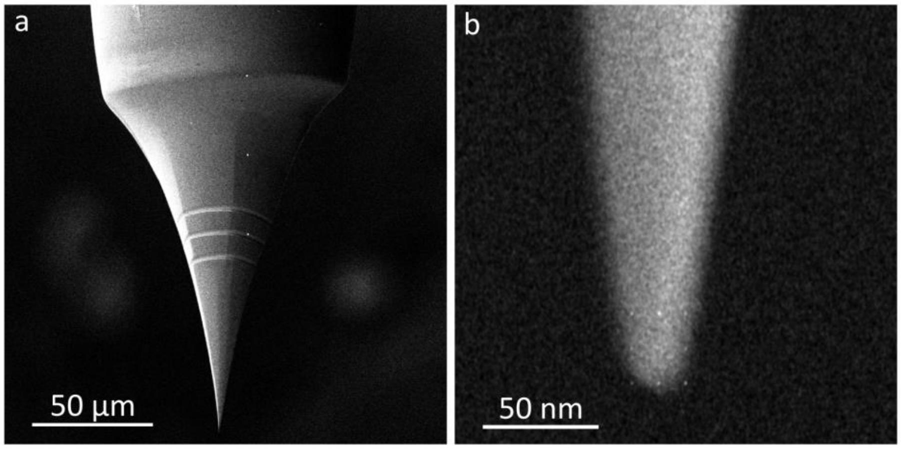

Figure S7. SEM images of the gold tip used as the photoelectron source. (a) The grating coupler is separated from the apex by ~80 µm. (b) The apex of the gold tip has an apex diameter of less than 30 nm.

5. Electron detection and data handling

The probe electrons are photo-emitted from the apex of the nanotip with a kinetic energy E_P. The electrons propagate along the z-direction towards the sample, placed at a distance of 5.8 µm from the tip. The distance is chosen to provide sufficiently high magnification of the electron microscope while preventing contributions from electrostatic field emission from the tip apex. Subsequently, they pass through the 176 mm long drift tube of the time-of-flight delay-line detector (ToF DLD, Hex80L by RoentDek Handels GmbH). The entrance of the drift tube is placed

at a distance of 10 mm from the sample. The nanotip, the sample and the drift tube are kept at the static potentials of $V_T < 0$, $V_S > 0$ and $V_D = 0$, with respect to ground. The potentials are set to $V_T = -80$ V and $V_S = +20$ V for the data shown in Fig. 2 and $V_T = -50$ V and $V_S = +30$ V for the data in Figs. 3 and 4. The bias voltage $U_{TS} = V_T - V_S$ accelerates the electrons and their kinetic energy in the sample plane is thus $E_0 = E_P - eU_{TS}$ (e : elementary charge, $E_0 \approx 100$ eV for the measurement in Fig. 2 and $E_0 \approx 80$ eV for those in Figs. 3 and 4). For the 13-nm thick film used in our experiments, a minimum bias of ~50 V is required. Working at an even lower bias voltage would allow for decreasing the tip sample distance to below a few μm, yet requires stronger localization of the near field to fulfill phase matching at reduced electron velocities.[2] The bias voltage between sample and drift tube $U_{SD} = V_S - V_D = V_S$ is set to a positive value and thus leads to a deceleration of the electrons. Their kinetic energy at the start of the drift tube is given by $E_1 = E_0 - eV_S$ ($E_1 \approx 80$eV (Fig. 2) and $E_1 \approx 50$ eV (Figs. 3 and 4)). Within the drift tube, the particles are shielded from stray electric and magnetic fields. At the end of the drift tube, the electrons arrive at a metal mesh with >75% transmission and are accelerated towards a 1 mm distant micro channel plate (MCP) that is kept at $V_{MCP} = 150$ V. The relative time difference between the arrival at the MCP and the arrival time of the laser pulse at the apex of the gold tip is taken as the time-of-flight (ToF) T_{TOF}. Behind the MCP, the impact position $\mathbf{r}_d = (x_d, y_d)$ of the electrons on the $x_d y_d$-detector plane is recorded with the delay-lines of the DLD with an accuracy of better than 200 µm[19]. The electron average velocity is then taken as $\mathrm{v}_0 = \sqrt{\mathbf{r}_d^2 + D^2} / T_{TOF}$, where D denotes the distance between sample and detector. The velocity component along the

tip-detector axis gives the momentum k_z as $k_z = mD/(\hbar T_{TOF})$ while the transverse momentum components are $k_x = x_d \cdot k_z / D$ and $k_y = y_d \cdot k_z / D$. The kinetic energy E of each individual electron is calculated as $E \approx \hbar^2 \mathbf{k}^2 / 2m = \hbar^2 (k_x^2 + k_y^2 + k_z^2)/2m$. The time T_{TOF} is on the order of a few tens of ns and is measured with an accuracy of better than 200 ps, resulting in a relative kinetic energy resolution of ~0.3 eV.

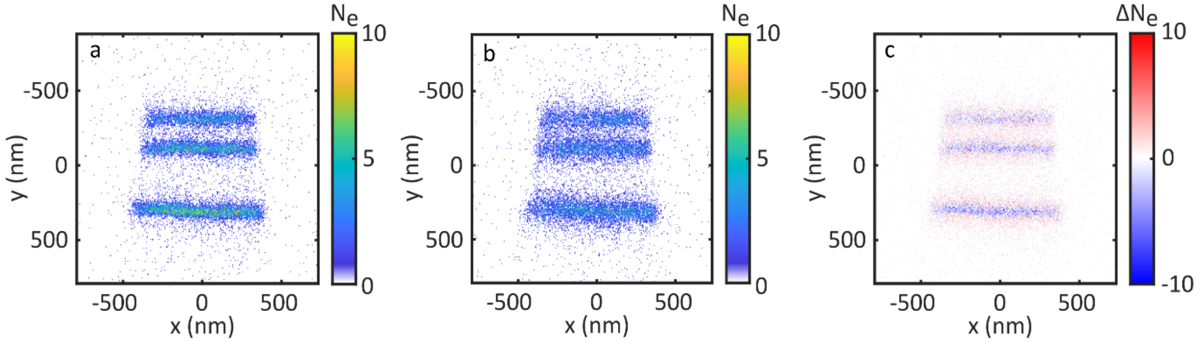

Figure. S8. UPEM image at a single delay step around delay time zero, for the measurement shown in Fig. 3 in the manuscript. The data are shown in the absence (a) and in the presence (b) of the optical pump pulse. (c) Difference between the image with and without pump pulse.

To perform a single measurement at a fixed delay time in our UPEM , the impact positions and kinetic energies of the probe electrons were accumulated over 200 s (Fig. 2), 20 s (Fig. 3) and 80 s (Fig. 4), with and without optical excitation of the sample. The delay step size was 15 fs for the measurement shown in Fig. 3 and 5 fs for that in Fig. 4, respectively. To create a two-dimensional image from the accumulated data, the impact positions $\mathbf{r}_d = (x_d, y_d)$ of the electrons with energies around the kinetic energy $E_1 \pm 10eV$ are used to fill a two dimensional histogram using a bin size of 200 µm. The positions are rescaled by the magnification M ~ 35.000, set by the ratio of detector sample and sample tip distance, to correspond to the impact position in the sample

plane $\mathbf{r} = (x, y) = \mathbf{r}_d / M$. This histogram, the number of detected electrons $N_e(x, y)$ at a certain position, is what we refer to as the UPEM image. The UPEM image for a single step around delay zero, corresponding to the data shown in Fig. 3d-f, is shown in Fig. S8.

Kinetic energy spectra are obtained by creating histograms of the probability $N_e(E)$ of detecting an electron at a certain energy E . Such histograms are recorded for each point across the complete detection area. Centered bins with a bin size of 0.3 eV were used. The histograms are shifted, using the known bias voltages, to reflect the kinetic energy at the sample plane. Some of the measurements shown in Fig. 3 reveal a slight background stemming from electrons that are emitted by DC field emission. This background is subtracted from the corresponding histograms, shown exemplary in Fig. S9. The resulting histograms are shown color-coded in Fig. 3a-b.

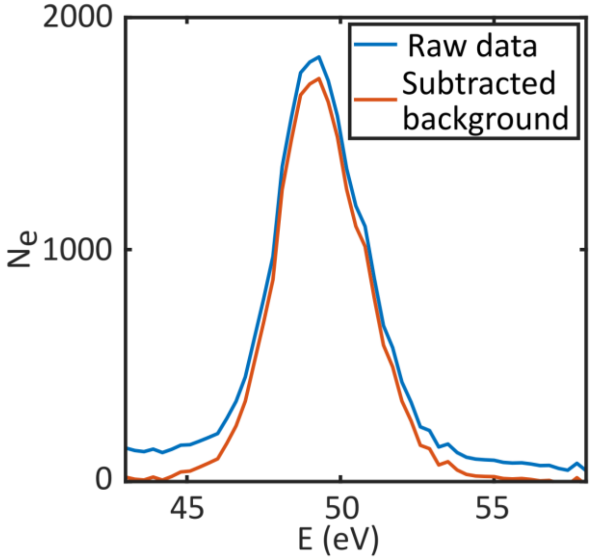

Figure. S9. Kinetic energy distribution $N_e(E)$ prior (blue line) and after (orange line) subtraction of the background signal.

6. Spatial resolution of the UPEM

The spatial resolution of the UPEM is estimated by analyzing the edge sharpness of the UPEM image. A cross-section of the UPEM image in Fig. 2a, taken along a line perpendicular to the edge of a single antenna arm, is displayed in Fig. S10 (blue circles). A fit to a function that is exponentially decaying with distance from the slit position is added as a red line. The fit reveals a 1/e decay length of ~20 nm. This resolution is on the order of the dimension of the apex of the gold tip that is used as the photoelectron source. It is certainly also affected by the finite thickness of the antenna sample. This high resolution and the virtually background-free imaging contrast convincingly demonstrate that the detected electrons are emitted from the very apex of the gold tip, confirming efficient plasmonic nanofocusing within the experiment.

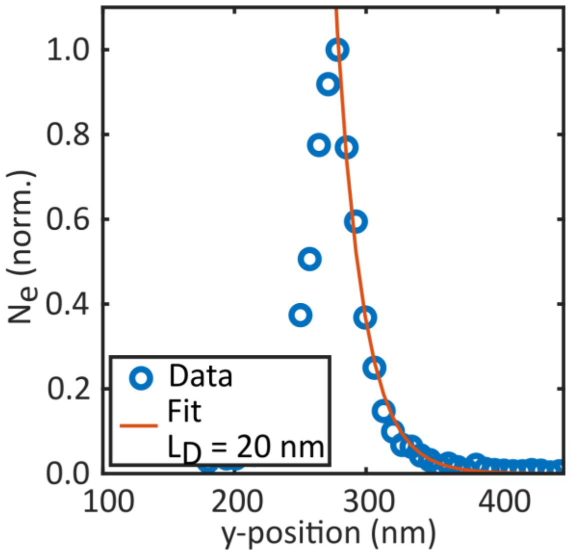

Figure S10. Crosscut through the UPEM image shown in Fig. 2 with an exponential decaying fit with a decay length of 20 nm.

7. Finite Difference Time Domain simulation

To gain insight into the field distribution in the vicinity of the Yagi-Uda antenna, finite difference time domain (FDTD) simulations were performed using the commercially available software package Lumerical (Ansys). The antenna structure is modelled by an array of three slits written in a 13-nm gold film, placed in the xy-plane at z = 0. The slits have a width of 40-nm in the y-direction and are oriented along the x-axis. The separation of the slits along the y-direction is 380 nm and 200 nm, respectively. The slits have lengths of ~500 nm (x-direction), slightly decreasing across the array. The geometry was chosen to reach local field enhancement factors larger than 10 inside the slits. The refractive index data for the gold film was taken as $n = 0.75 + i \cdot 12$ and the region closely around the antenna slits was meshed at a fixed step size of 2nm. The source is a plane-wave source centered at $\omega_p = 0.993$ PHz (~1900 nm) with 20-fs temporal duration. At this wavelength, we resonantly excite the lowest-order symmetric eigenmode of the antenna. The wave is linearly p-polarized along the yz-plane and propagates towards the sample under an angle of incidence of $\Theta = 58°$. The borders of the simulated region are modelled as perfectively absorbing layers (PML). We confirmed that a further increase in size of the simulation region did not affect the results. We monitor the electric field in the xy-plane at the center of the film (Fig. S11). Importantly, the E_y component, pointing perpendicular to the slit axis, is strongly enhanced inside the slits, showing local field enhancements that exceed 10 across the entire width of the slits. All other field components are much weaker. Since the film is thin, the field enhancement for E_y is basically constant across the thickness of the film. This

makes the structure interesting for studying the near-field deflection of slow electrons, as demonstrated experimentally.

In the Lumerical simulations, the components in z-direction, E_z, are mostly enhanced in the direct vicinity of the sharp edges of the slits. The localization length is mainly defined by the chosen mesh size. In contrast, the nano-fabricated slit structures have finite radii of curvature at their edges, limited by the physical properties of the helium ion beam that is used to mill the structures. For the used Zeiss Orion helium ion beam microscope the radius of curvature is on the order of 6 nm[20]. Hence the field distribution that is generated by the dipole model in Fig. S1 appears to be a more realistic modelling of the z-components of the field. This conclusion is further supported by the reasonable agreement between experiment and simulations, using the dipole field model, that is shown in Fig. 4a,b. Thus, we have used the fields from the dipole model shown in Fig. S1 to compare experimental and simulated kinetic energy spectra. No further attempts were made to improve the FDTD simulations by decreasing the edge sharpness.

The time dependence of the electric field amplitudes E_y and E_z at the center of the left most antenna slit is shown in Fig. S12. Evidently the local field dynamics are very similar to those of the incident field. More systematic wavelength-dependent studies of the field enhancement suggest that the lowest order eigenmode of the antenna is centered around 2000 nm and has a lifetime of less than 5 fs. Thus, the time dynamics observed in our optical pump – electron probe measurements remain unaffected by the finite lifetime of the antenna resonance.

Since the experimental data has been recorded for two different orientations of the antenna with respect to the incident beam, we display, in Fig. S13, the maximum electric field amplitude across

the xy-plane for these two orientations. Upon rotation of the sample only the position of the highest field amplitude inside the array changes. We assign this to the interference between the external light field and plasmon fields launched at the slits.[21] This explains why the near field induced electron deflection shown in y Fig. 3 is largest for the bottom slit (Fig. S13b) but more pronounced at the two closely slits in the measurement shown in Fig. 2 (Fig. S13a). The correlation between the illumination condition, local field enhancement and experimentally observed deflection pattern, indicates that our measurement directly maps the near field distribution of the Yagi-Uda antenna. The spatially homogeneous field enhancement of the E_y component of the order of 10 inside the slits nicely accounts for the spatial dependence of the observed near field electron deflection in *y* - direction.

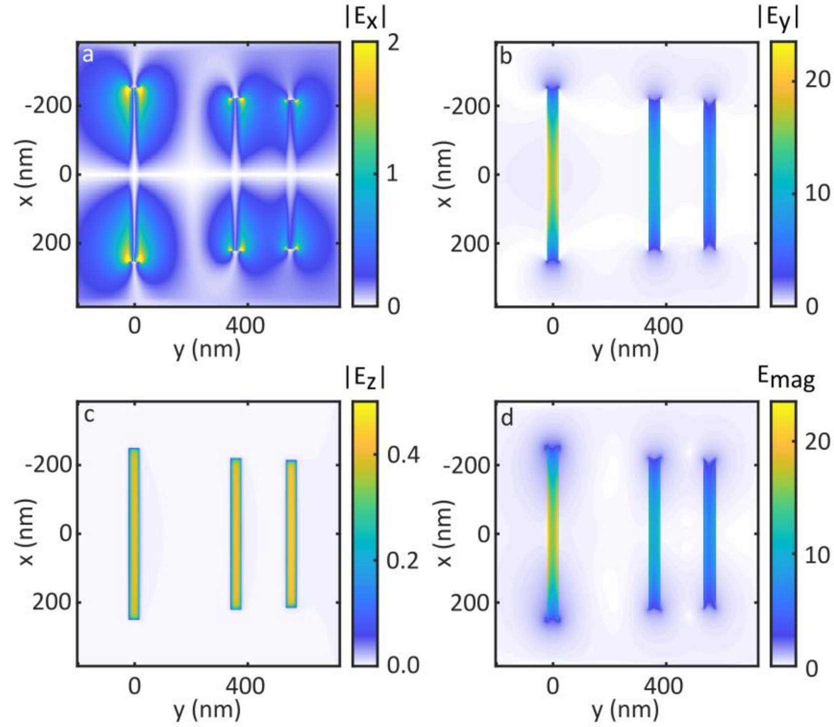

Figure S11. Maximum electric field amplitudes as a function along the xy-plane at z=0 (center of the gold film). The maximum field enhanced inside the slit antenna is ~ 20.

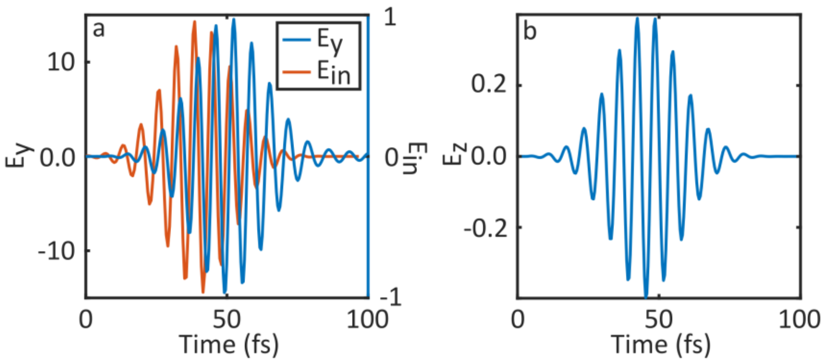

Figure. S12. (a) Time-structure of the incident electric field $E_{in}(t)$ (red line) and the y-component of the local field, $E_y(\mathbf{r}=0,t)$ (blue line), at the center of the antenna. (b) Time structure of $E_z(\mathbf{r}=0,t)$ (blue line). The time structure of local field follows the incident field, demonstrating the quasi-instantaneous response of the slit antenna.

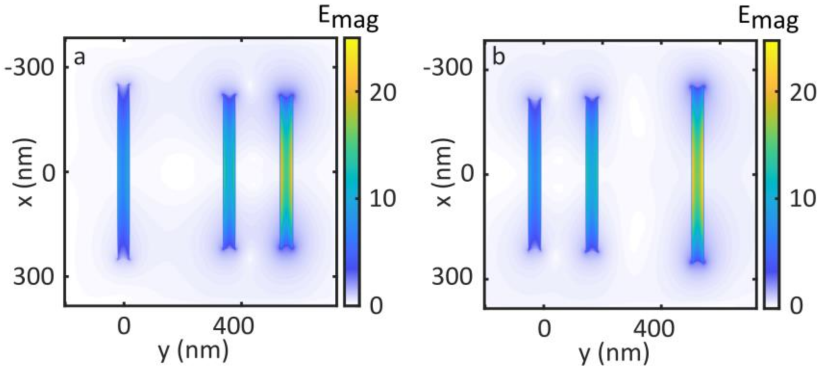

Figure. S13. Maximum electric field amplitudes across the antenna for two different orientations of the Yagi-Uda antenna with respect to the incident field.

8. Chirped electron wavepackets

We first consider an electron wavepacket as described by Eq.(4), propagating solely in z-direction. Upon free-space propagation the wavepacket envelope accumulates a quadratic phase $\varphi_k(k_z)$ in momentum space (see Eq.(10)):

$$\tilde{g}(k_z,t)=\tilde{g}(k_z,0)\exp\left(-i\frac{\hbar k_z^2}{2m}t\right)=\tilde{g}(k_z,0)\exp\left(i\varphi_k(k_z)\right) \qquad (28)$$

The quadratic phase $\varphi_k(k_z)$ results in a quadratic phase in real space $\varphi_r(z)$ that can be transferred to a quadratic phase in time $\varphi_t(t)$ by the electrons average velocity $t = z / v_0$. To characterize this phase, we define an instantaneous kinetic energy E_I of the electron as $E_I = \hbar\left(\omega_0 - \frac{\partial\varphi_t(t)}{\partial t}\right)$. In Fig. S14a, we display E_I as a function of time for an initially bandwidth-limited electron wavepacket with a central kinetic energy of $E_0 = 80$ eV and width $\Delta E =$ 3 eV after a propagation time of t = 2 ps. A down chirp of the electron wavepacket is evident (Fig. S14a). This chirp is characterized for the chirp of the electron wavepacket acquired upon propagation from tip to sample.

When simulating the interaction of such a chirped electron wavepacket with the near-field potential depicted in Fig. S1, a chirp-induced shift in the delay-dependent kinetic energy distribution is clearly seen (Figs. 4b and S14b). In those simulations the near-field potential is modeled as $\Phi(y,z,t) = \int_{-\infty}^{\infty} \Phi_G(y,z,\omega)\cos(\omega t)d\omega$ with $\Phi_G(y,z,\omega) = G\left(\omega - \omega_p\right)\Phi_0(y,z)$. The Gaussian G describes the spectrum of the employed optical pump. In the simulation shown in Fig. 4b the FWHM of G is set to 0.2 PHz, resulting in a pulse duration of roughly 20 fs and the duration of the electron wavepacket is set to 50 fs duration by free-space propagation, in agreement with expectations, assuming an initial electron pulse duration of 10-20 fs together with a ~20 fs dispersive broadening of the electron pulses during the 1-ps propagation from the tip to the 5.8 µm distant sample. In the simulation shown in Fig. S14b the duration of the optical pump was reduced to 10 fs and the electron pulse duration was set to 40 fs to show the effect of the near-field interaction more clearly. Here, the magnitude of the slope of this energy shift of

0.08 eV/fs (indicated by the black line in Fig. S14b) is the same as the slope of E_I for the incident wavepacket (Fig. S14a). The sign is reversed since fast electrons arrive early but are deflected at positive delays. The absolute value of this slope is inversely proportional to the pulse duration of the chirped electron pulse in the sample plane (Fig. S14c). The value of 0.025 eV/fs deduced from Fig. 4 is measured for an optical pulse duration of 20 fs and agrees reasonably well with the results of the simulations presented in Fig. S14c.

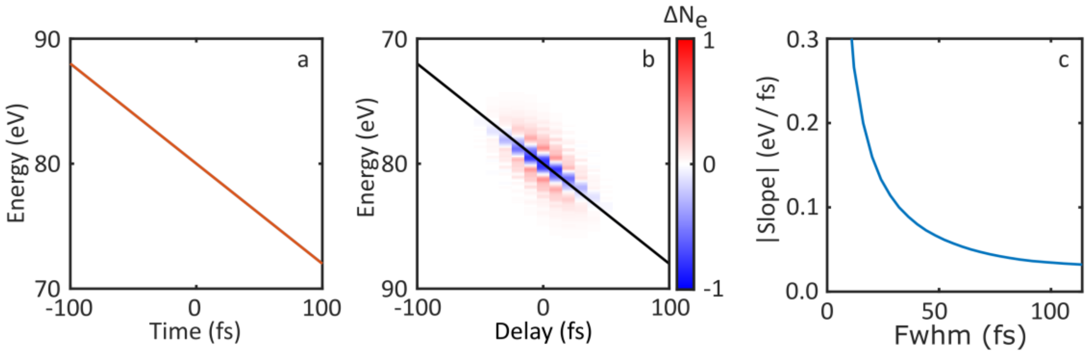

Figure S14. (a) Instantaneous kinetic energy E_I of an initially bandwidth limited electron wavepacket with energy $E_0 = 80$ eV and $\Delta E =$ 3 eV after 2 ps propagation in free space. The dispersive propagation results in a down-chirp of the electron wavepacket and an increase in its temporal duration to ~40 fs. (b) Simulations of the kinetic energy distribution of such a 40-fs electron wavepacket passing through the near-field of a Yagi-Uda antenna (Fig. S1), generated by a 10-fs optical pump pulse at 1900 nm. The slope of 0.08 eV/fs seen in the delay dependent energy distribution matches the absolute value of the slope of E_I in (a), indicated by the black line in (b). (c) Chirp-induced slope of the kinetic energy distribution seen in (b) for different pulse durations of the electron wavepacket in the sample plane. The pulse duration of the optical pump is fixed to 10 fs.

9. Spatial localization and interaction time

Phase matching between near field and electrons requires that the electron transit time T through the optical near field less than half an optical cycle (3.2 fs at 1900 nm, $\omega_p \approx 1$ PHz). Otherwise

field oscillations will start to average out the effect of the near field on the probe electrons. Evidently, this puts rather stringent requirement on the spatial field localization Δz that is needed to achieve phase matching with slow electrons. In Fig. S15, the required localization length is shown as a function of the electron kinetic energy on a double logarithmic scale. The needed localization is given as $\Delta z = \pi/\Delta k = v_e \cdot \pi/\omega_p$, the product of the electron velocity and the half cycle time of the optical near field.

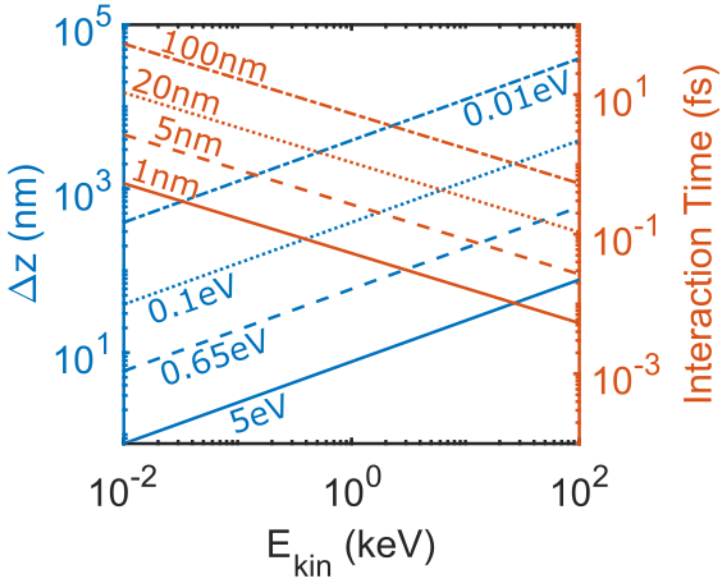

Figure S15. Phase matching with slow electrons. Localization length Δz of the optical near field that is needed to achieve optimum phase matching, i.e. an interaction time between electrons and near field of less than an optical half cycle, in dependence on the electron kinetic energy (blue lines) for different photon energies of 0.01 eV (dashed-dotted), 0.1 eV (dotted), 0.65 eV (dashed) and 5 eV (solid). Additionally, the interaction time between near-field and electrons is given (red lines) for different localization lengths Δz between 1 and 100 nm as a function of kinetic energy All data are shown on double logarithmic scales.

In Fig. S15, Δz is shown for four different photon energies $\hbar\omega_p$ of 0.01 eV (λ≈100 µm), 0.1 eV (λ≈10 µm), 0.65 eV (λ≈1900 nm) and 5 eV (λ≈250 nm). It is evident that, e.g. for 0.65 eV (dashed

blue line) the requirement of the spatial localization of 20 nm, for electron energies around 100 eV, is much harder to fulfill than for swift electrons around 100 keV where phase matching is fulfilled even for a spatial extent of the near field of more than 500 nm. On the right axis, the interaction time between electrons and near field is shown as a function of the kinetic energy for different localization lengths to elucidate the increased interaction time at lower electron velocities.

References

1. Park, S. T.; Lin, M.; Zewail, A. H. Photon-induced near-field electron microscopy (PINEM): theoretical and experimental. *New Journal of Physics* **2010,** 12, (12).
2. Hergert, G.; Wöste, A.; Groß, P.; Lienau, C. Strong inelastic scattering of slow electrons by optical near fields of small nanostructures. *Journal of Physics B: Atomic, Molecular and Optical Physics* **2021,** 54, (17).
3. Herink, G.; Solli, D. R.; Gulde, M.; Ropers, C. Field-driven photoemission from nanostructures quenches the quiver motion. *Nature* **2012,** 483, (7388), 190-3.
4. Park, D. J.; Piglosiewicz, B.; Schmidt, S.; Kollmann, H.; Mascheck, M.; Lienau, C. Strong field acceleration and steering of ultrafast electron pulses from a sharp metallic nanotip. *Phys Rev Lett* **2012,** 109, (24), 244803.
5. Piglosiewicz, B.; Schmidt, S.; Park, D. J.; Vogelsang, J.; Groß, P.; Manzoni, C.; Farinello, P.; Cerullo, G.; Lienau, C. Carrier-envelope phase effects on the strong-field photoemission of electrons from metallic nanostructures. *Nature Photonics* **2014,** 8, (1), 37-42.
6. Dombi, P.; Pápa, Z.; Vogelsang, J.; Yalunin, S. V.; Sivis, M.; Herink, G.; Schäfer, S.; Groß, P.; Ropers, C.; Lienau, C. Strong-field nano-optics. *Reviews of Modern Physics* **2020,** 92, (2).
7. Feist, A.; Bach, N.; Rubiano da Silva, N.; Danz, T.; Möller, M.; Priebe, K. E.; Domröse, T.; Gatzmann, J. G.; Rost, S.; Schauss, J.; Strauch, S.; Bormann, R.; Sivis, M.; Schäfer, S.; Ropers, C. Ultrafast transmission electron microscopy using a laser-driven field emitter: Femtosecond resolution with a high coherence electron beam. *Ultramicroscopy* **2017,** 176, 63-73.
8. Dahan, R.; Nehemia, S.; Shentcis, M.; Reinhardt, O.; Adiv, Y.; Shi, X.; Be'er, O.; Lynch, M. H.; Kurman, Y.; Wang, K.; Kaminer, I. Resonant phase-matching between a light wave and a free-electron wavefunction. *Nature Physics* **2020,** 16, (11), 1123-1131.
9. Vanacore, G. M.; Madan, I.; Berruto, G.; Wang, K.; Pomarico, E.; Lamb, R. J.; McGrouther, D.; Kaminer, I.; Barwick, B.; Garcia de Abajo, F. J.; Carbone, F. Attosecond coherent control of free-electron wave functions using semi-infinite light fields. *Nat Commun* **2018,** 9, (1), 2694.
10. Feist, A.; Echternkamp, K. E.; Schauss, J.; Yalunin, S. V.; Schafer, S.; Ropers, C. Quantum coherent optical phase modulation in an ultrafast transmission electron microscope. *Nature* **2015,** 521, (7551), 200-3.
11. Manzoni, C.; Cerullo, G. Design criteria for ultrafast optical parametric amplifiers. *Journal of Optics* **2016,** 18, (10).
12. Neuhaus, M.; Fuest, H.; Seeger, M.; Schotz, J.; Trubetskov, M.; Russbueldt, P.; Hoffmann, H. D.; Riedle, E.; Major, Z.; Pervak, V.; Kling, M. F.; Wnuk, P. 10 W CEP-stable few-cycle source at 2 microm with 100 kHz repetition rate. *Opt Express* **2018,** 26, (13), 16074-16085.
13. Stibenz, G.; Steinmeyer, G. Interferometric frequency-resolved optical gating. *Opt. Express* **2005,** 13, (7), 2617-2626.

14. Zhong, J. H.; Vogelsang, J.; Yi, J. M.; Wang, D.; Wittenbecher, L.; Mikaelsson, S.; Korte, A.; Chimeh, A.; Arnold, C. L.; Schaaf, P.; Runge, E.; Huillier, A. L.; Mikkelsen, A.; Lienau, C. Nonlinear plasmon-exciton coupling enhances sum-frequency generation from a hybrid metal/semiconductor nanostructure. *Nat Commun* **2020,** 11, (1), 1464.
15. Trebino, R.; Kane, D. J. Using phase retrieval to measure the intensity and phase of ultrashort pulses: frequency-resolved optical gating. *J. Opt. Soc. Am. A* **1993,** 10, (5), 1101-1111.
16. Vogelsang, J.; Robin, J.; Nagy, B. J.; Dombi, P.; Rosenkranz, D.; Schiek, M.; Gross, P.; Lienau, C. Ultrafast Electron Emission from a Sharp Metal Nanotaper Driven by Adiabatic Nanofocusing of Surface Plasmons. *Nano Lett* **2015,** 15, (7), 4685-91.
17. Schmidt, S.; Piglosiewicz, B.; Sadiq, D.; Shirdel, J.; Lee, J. S.; Vasa, P.; Park, N.; Kim, D.-S.; Lienau, C. Adiabatic Nanofocusing on Ultrasmooth Single-Crystalline Gold Tapers Creates a 10-nm-Sized Light Source with Few-Cycle Time Resolution. *ACS Nano* **2012,** 6, (7), 6040-6048.
18. Vogelsang, J.; Talebi, N.; Hergert, G.; Wöste, A.; Groß, P.; Hartschuh, A.; Lienau, C. Plasmonic-Nanofocusing-Based Electron Holography. *ACS Photonics* **2018,** 5, (9), 3584-3593.
19. Jagutzki, O.; Cerezo, A.; Czasch, A.; Dorner, R.; Hattas, M.; Min, H.; Mergel, V.; Spillmann, U.; Ullmann-Pfleger, K.; Weber, T.; Schmidt-Bocking, H.; Smith, G. D. W. Multiple hit readout of a microchannel plate detector with a three-layer delay-line anode. *IEEE Transactions on Nuclear Science* **2002,** 49, (5), 2477-2483.
20. Kollmann, H.; Piao, X.; Esmann, M.; Becker, S. F.; Hou, D.; Huynh, C.; Kautschor, L. O.; Bosker, G.; Vieker, H.; Beyer, A.; Golzhauser, A.; Park, N.; Vogelgesang, R.; Silies, M.; Lienau, C. Toward plasmonics with nanometer precision: nonlinear optics of helium-ion milled gold nanoantennas. *Nano Lett* **2014,** 14, (8), 4778-84.
21. Kubo, A.; Pontius, N.; Petek, H. Femtosecond Microscopy of Surface Plasmon Polariton Wave Packet Evolution at the Silver/Vacuum Interface. *Nano Letters* **2007,** 7, (2), 470-475.
